# Dynamic Imaging of the Effector Immune Response to *Listeria* Infection *In Vivo*

**DOI:** 10.1371/journal.ppat.1001326

**Published:** 2011-03-24

**Authors:** Janelle C. Waite, Ingrid Leiner, Peter Lauer, Chris S. Rae, Gaetan Barbet, Huan Zheng, Daniel A. Portnoy, Eric G. Pamer, Michael L. Dustin

**Affiliations:** 1 Program in Molecular Pathogenesis, Helen L. and Martin S. Kimmel Center for Biology and Medicine of the Skirball Institute of Biomolecular Medicine, New York University School of Medicine, New York City, New York, United States of America; 2 Infectious Disease Service, Department of Medicine, Memorial Sloan-Kettering Cancer Center, Immunology Program, Sloan-Kettering Institute, New York City, New York, United States of America; 3 Aduro BioTech, Berkeley, California, United States of America; 4 Department of Molecular and Cell Biology, University of California, Berkeley, Berkeley, California, United States of America; 5 Department of Chemical Engineering, Massachusetts Institute of Technology, Cambridge, Massachusetts, United States of America; 6 School of Public Health, University of California, Berkeley, Berkeley, California, United State of America; Stanford University, United States of America

## Abstract

Host defense against the intracellular pathogen *Listeria monocytogenes* (*Lm*) requires innate and adaptive immunity. Here, we directly imaged immune cell dynamics at *Lm* foci established by dendritic cells in the subcapsular red pulp (scDC) using intravital microscopy. Blood borne *Lm* rapidly associated with scDC. Myelomonocytic cells (MMC) swarmed around non-motile scDC forming foci from which blood flow was excluded. The depletion of scDC after foci were established resulted in a 10-fold reduction in viable *Lm*, while graded depletion of MMC resulted in 30–1000 fold increase in viable *Lm* in foci with enhanced blood flow. Effector CD8^+^ T cells at sites of infection displayed a two-tiered reduction in motility with antigen independent and antigen dependent components, including stable interactions with infected and non-infected scDC. Thus, swarming MMC contribute to control of *Lm* prior to development of T cell immunity by direct killing and sequestration from blood flow, while scDC appear to promote *Lm* survival while preferentially interacting with CD8^+^ T cells in effector sites.

## Author Summary

The pathogenic bacteria *Listeria monocytogenes* (*Lm*) can access the intracellular space and establish infection in the host spleen and liver. Innate immune cells are required to detect and contain infection while specific adaptive T cells are activated and recruited to infection sites. Adaptive immunity enhances the function of innate cells and also directly kills infected cells to prevent further bacterial propagation. These cells respond to local environmental cues and often communicate via direct cell-to-cell contact. Here, we observed cellular dynamics *in situ* by imaging live tissue using intravital microscopy. We reconstituted the *Lm* infection model with bacteria and mouse strains engineered to express fluorescent proteins to label specfic cells. We find that dendritic cells (DC) in the spleen rapidly capture *Lm* from circulation, demonstrating a direct role of these cells in sequestering bacteria. Neutrophils converge on infected cells by directed migration to generate exanguinous foci within the otherwise blood rich red pulp. Finally, antigen specific CD8^+^ T cell interact extensively with infected cells and DC in the foci and are selectively retained compared to non-specific polyclonal T cells. These findings significantly advance our understanding of immune cell dynamics in the spleen in both the steady state and in response to *Listeria* infection. These studies could aid in development of disease therapies and vaccine strategies targeted towards enhancing DC, myelomonocytic and CD8^+^ T cell effector responses.

## Introduction

Appropriate host immune response to pathogenic invasion is critical for survival. Secondary lymphoid tissues provide a structural context for multiple layers of the innate and adaptive immune response to infection. The spleen is a physiologically relevant tissue for immune responses to blood borne pathogens. The immune response to systemic *Listeria monocytogenes* (*Lm*) infection has been studied extensively in mice and is focused in the spleen and liver [1]. In this model, the innate immune system is responsible for detecting and containing infection while adaptive immunity is required for clearance of *Lm* and enhanced protection against future infections (memory) [2]. *Lm* is a gram positive intracellular bacterium and can cause severe infection in immune compromised individuals [3]. *Lm* expresses several virulence factors that enable invasion of the cytoplasm and movement from cell to cell through a contact dependent mechanism and thus grows in foci established by infection of one cell [4].

The spleen acts as a blood filter with a network of phagocytic cells in the marginal zone (MZ) and red pulp (RP) that are in direct contact with 5% of the the cardiac output. CD11c^+^ dendritic cells (DC) in the MZ and RP of the spleen sequester *Lm* from the blood and are required to initiate infection in the spleen [5], [6], [7]. At the site of infection DC orchestrate both recruitment and activation of innate effectors by secretion of inflammatory cytokines [8]. Aggregations of immune cells including myelomomonocytic cells (MMC) consisting of neutrophils and inflammatory monocyte subsets at foci of *Lm* growth are required to restrict bacterial growth and contain infected cells [9], [10], [11]. The process through which MMC converge on foci in the spleen is not understood. For example, expression of chemokine receptor CCR2 is required for monocyte egress from the bone marrow, but is dispensable for homing to infection sites once in the blood [12].

Although the innate immune system can restrict *Lm* infection, cells of the adaptive system, particularly CD8^+^ T cells, are required for sterilizing immunity [13]. DC bearing *Lm* antigens migrate from the MZ to the white pulp (WP) in a pertussis toxin sensitive process where they present antigen to T cells, which is required to prime adaptive immune responses [14], [15], [16]. DC that prime CD8^+^ T cells may be directly infected or acquire *Lm* antigens from infected apoptotic cells such as neutrophils [17], [18], [19]. After activation, CD8^+^ T cells proliferate extensively and exit the WP for bacterial clearance in the RP and to gain access to other sites of infection through the blood [20]. The mechanism of clearance is not completely understood, but requires perforin, IFNγ, TNFα and CCL3 [21], [22]. Direct visualization of this process may provide insight as *in vivo* cytotoxicity has been associated with stable and prolonged interactions of antigen specific cytotoxic T lymphocytes (CTL) and target cells [23], [24]. Tissue DC also play a role in the effector phase of T cell responses providing local signals for cytokine production [25], [26], [27], [28]. However, the role of tissue DC in clearance of *Lm* and nature of T-DC interactions in RP foci is unknown.

Live imaging of lymphoid structures by intravital microscopy has provided high-resolution information on the dynamic interactions that take place during immune responses *in situ*. Tracking of cells in real time reveals kinetic information that is lacking from static images. Intravital microscopy has been useful in understanding tissue specific responses to pathogens [29]. These studies have revealed active environmental sampling by resident DC networks that act as sentinals in peripheral organs such as skin and intestines during infection by Salmonella in the gut and protozoan parasites in the skin [30], [31], [32], [33]. In addition, patrolling neutrophils and monocytes rapidly respond to invading *Leishmania major* or tissue injury [34], [35], [36]. The dynamics of these cells at inflammatory lesions in zebrafish models of tuberculosis have yielded unexpected role of macrophages in dissemination of infection [37]. In mouse models of *Leishmania donovani* and Bacillus Calmette-Guérin (BCG) infection, T cells are rapidly recruited to liver granulomas by inflammatory signals and antigen specific cells are retained [38], [39]. Interactions of effector T cells with parasites and resident antigen presenting cells (APC) revealed zones of antigen specific contacts with pathogen associated APC, while some infected cells were not contacted by T cells in the brain and in the skin [40], [41], [42]. The mode of interaction of *Lm* specific effector T cells in infectious foci is unknown and cannot be predicted based on existing data.

Multiphoton intravital imaging in the spleen is hindered by light absorption and scattering by red blood cells and auto-fluorescent metabolites concentrated in the RP. This interference is avoided by directly imaging WP of spleen fragments *ex vivo* [43], [44]. However with these preparations physiological circulation and recruitment of cells from hematopoietic tissues such as the bone marrow are lost. We have found that intravital imaging in the RP with confocal microscopy yields high-resolution images suitable for analyzing cellular dynamics. We have previously reported the presence of an extensive DC network in the subcapsular RP (scDC) that can be directly visualized by intravital microscopy of the spleen [45]. Early after infection, macrophages and DC capture the bulk of *Lm* in the MZ and then migrate to the WP to present *Lm* antigens to activate T cells [5], [6], [16], [46]. However, a minority of *Lm* does access the RP and establish foci of bacterial growth there. In addition, T cells exit the WP after activation to clear infected cells in the RP [20]. The role of the scDC network and immune cell dynamics in the response to *Lm* infection in the RP is unknown. Here we set out to observe the early events in the innate immune response and later clearance of *Lm* infection by adaptive T cells using *in vivo* imaging of the RP. We show that scDC interacted with *Lm* within 2 minutes after introduction into the blood stream, but at 48–72 hours post infection (p.i.) did not directly contribute to the innate control of bacterial growth once *Lm* foci are established. At 48 hours p.i., MMC converged on foci by directed migration and restricted blood flow around infected cells. On day 5 p.i., the peak of *Lm* growth, CD8^+^ T cells are recruited to these foci and displayed a two-tiered deceleration with antigen independent and antigen dependent components.

## Results

### Systemic *Lm* is rapidly associated with subcapsular DC in the spleen red pulp

**Figure 1:**
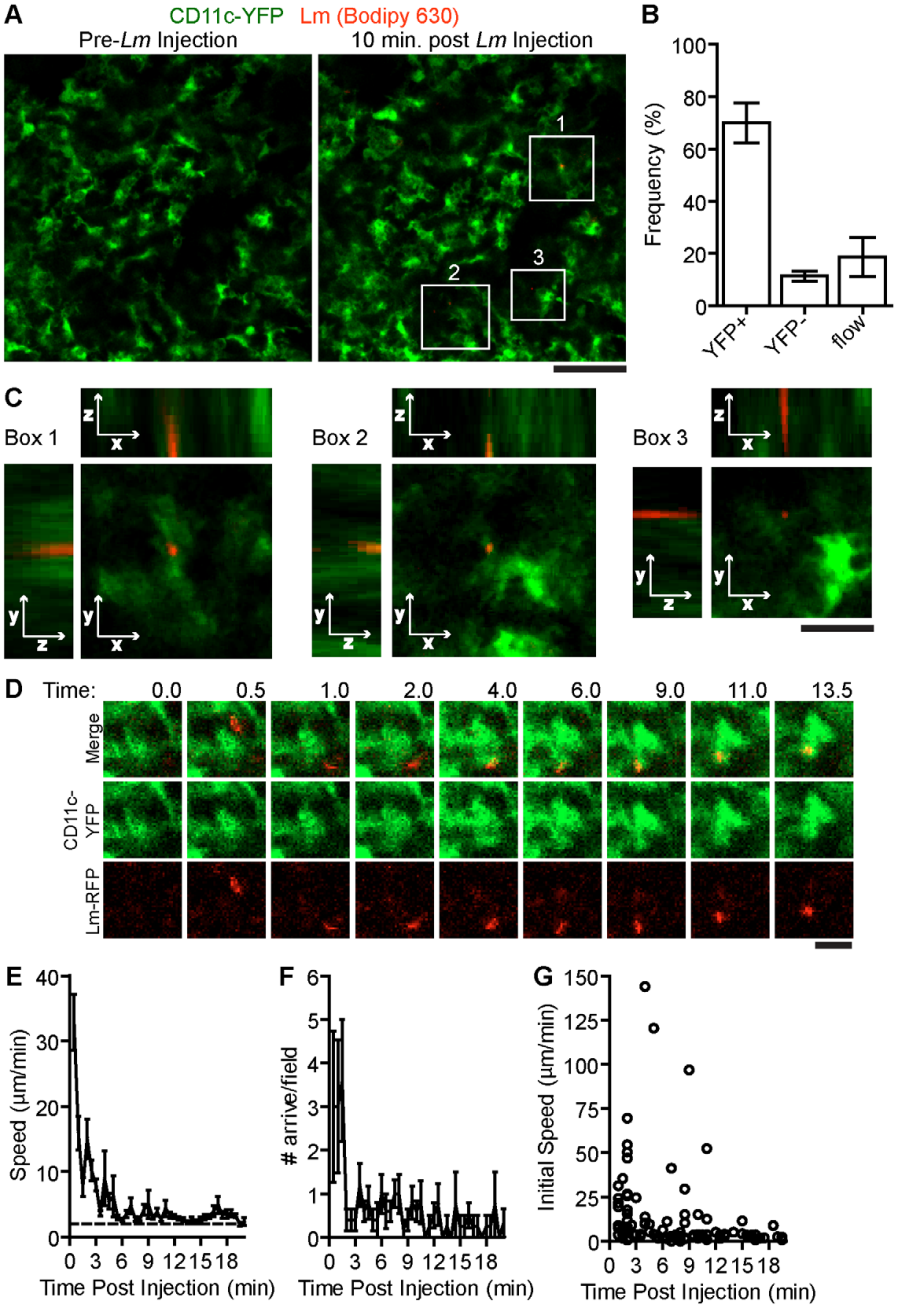
Systemic *Lm* rapidly associates with sinusoidal DC in the spleen RP. 10^7^ Bodipy-630 labeled *Lm* were injected i.v. while acquiring images in the spleen of CD11c-EYFP mice. A. Intravital snapshots of labeled *Lm* (red) and YFP^+^ DC (green) just before and 10 minutes after *Lm* injection. White boxes indicate *Lm* associated with DC. Scale bar = 50 µm. B. Frequency of *Lm* associated with YFP^+^ cells, YFP^−^ cells or in blood flow (in flow) within the imaging field. C. Intravital Z-stack images taken at 1 µm steps through *Lm* associated with DC shown in A (white boxes). Scale bar = 17 µm. D. Intravital images of CD11c-EYFP and *Lm* at the indicated times post injection (minutes). Scale bar = 10 µm. E. Mean speed (µm/min) of bacteria entering the field over time (minutes). F. Number of bacteria arriving in the imaging field over time (minutes). G. Instantaneous speeds of bacteria over time (minutes). Time is relative to the *Lm* injection. Data represents the mean from 135 bacteria from 6 mice. Error bars represent SEM.

To test if scDC take up *Lm*, we used intravital microscopy in the spleen subcapsular RP to image the arrival of fluorescently labeled *Lm* in circulation of CD11c-EYFP mice [47]. Frozen spleen sections from CD11c-EYFP mice show that YFP^+^ cells are present in the RP and these cells are heterogeneous for surface staining of CD11c, MHC class II, F4/80 and CD11b (Figure S1 in Text S1). In the subcapsular RP, YFP^+^ cells stained strongly for F4/80, a marker for RP macrophages but also expressed by a subset of DC isolated from spleen and DC subsets found in peripheral organs such as skin [25], [48]. Most of these subcapsular YFP^+^ cells also displayed dendritic morphology and time-lapse images of these cells show they are largely non-motile but are actively extending and retracting their dendrites (Figure 1A, Video S1). This activity is similar to other reports of environmental sampling by DC networks in the lymph node [47]. Thus, it seems that a majority of these YFP^+^ cells in the subcapsular RP represent a subset of peripheral tissue DC.

During image acquisition, 10^7^ Bodipy-630 labeled *Lm* were injected into the retro-orbital plexus (Video S1). In some experiments, *Lm* were injected together with a 10 kDa rhodamine dextran, to mark the time of injection. *Lm* arrived in the RP within 30 seconds of the rhodamine dextran arrival which was about 60 seconds post injection (data not shown). Of the *Lm* that came through the imaging field, 70+7.7% (n = 6 mice, 135 bacteria,) were associated with non-motile scDC 10 minutes after injection (Figure 1B.). *Lm*-associated scDC remained non-motile for the duration of image acquisition (up to 20 minutes) and continued to actively probe the environment (Video S1). High-resolution z-stack images show that *Lm* are primarily located within or at the periphery of scDC (Figure 1C). *Lm* were also co-localized with scDC in images of fixed spleen sections (Figure S1 in Text S1). The peripheral association may be due to the location of early phagocytic compartments, *Lm* attaching to or within dendrites below the limit of detection or via interactions with non-fluorescent cells containing *Lm*. By tracking *Lm* in the spleen *in vivo*, we found *Lm* speeds were initially high in the first few minutes post injection representing bacteria in flow however decelerated as they came in contact with DC (Figure 1D–E). The number of bacteria arriving in the spleen decreased to less than 1 per field after 2 minutes suggesting they are rapidly taken out of circulation (Figure 1F). The speeds of *Lm* that arrived at later time points were slower than those in flow suggesting they were within a slow moving non-fluorescent cell (Figure 1G).

### Patrolling neutrophils scavenge extracellular *Lm*

**Figure 2:**
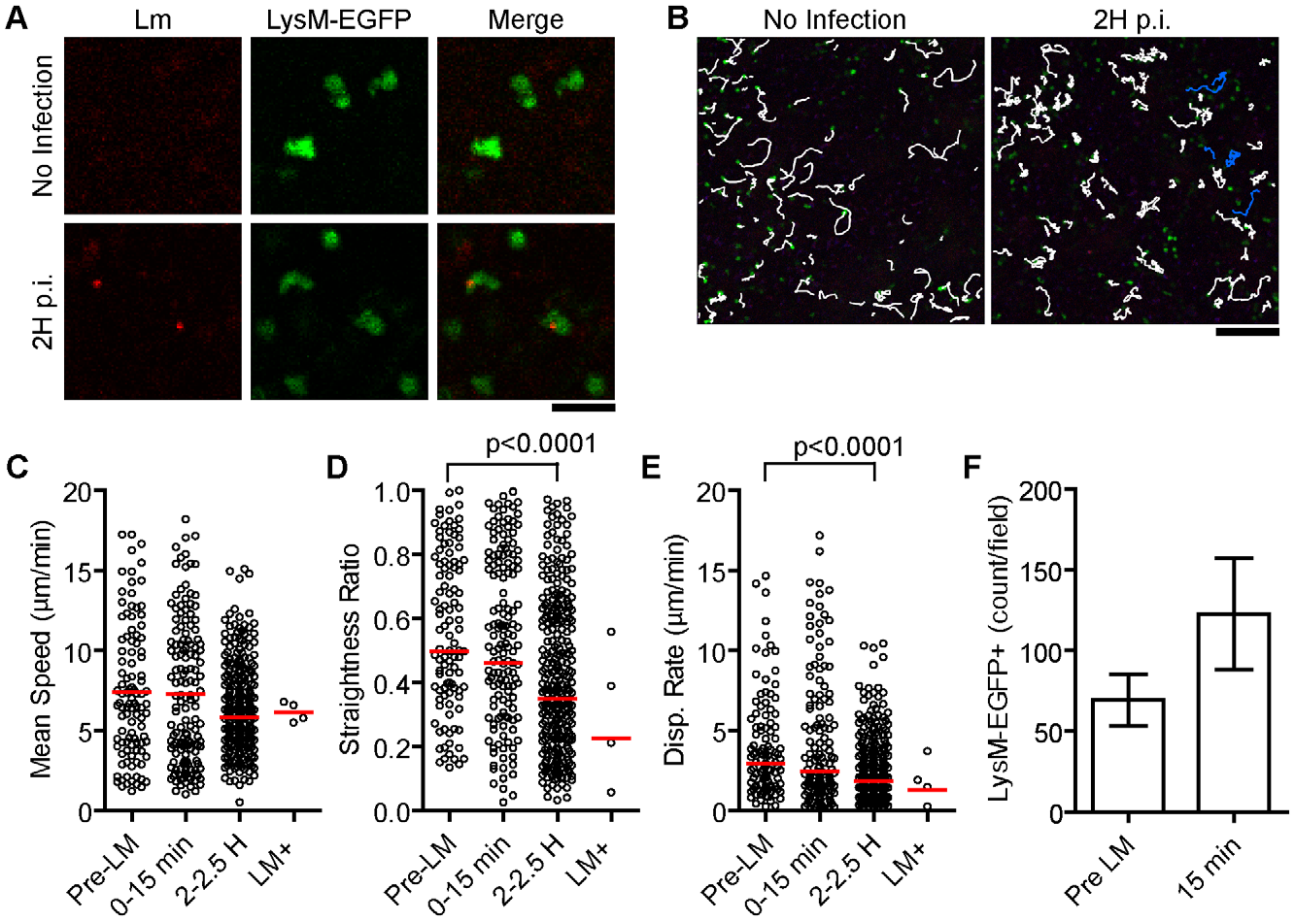
A small fraction of patrolling neutrophils take up *Lm*. 10^7^ Bodipy-630 labeled *Lm* were injected i.v. while acquiring images in the spleen of LysM-EGFP mice. A. Intravital snapshots of LysM-EGFP (green) and labeled *Lm* (red). Scale bar = 25 µm. B. Snapshots overlaid with neutrophil tracks (white) and *Lm*+ neutrophil tracks (blue). Scale bar = 100 µm. C–E. Motility parameters before (Pre-LM), acutely after (0–15 min), and 2 hours after (2–2.5 H) *Lm* injection separating out neutrophils that took up *Lm* at 2 hours post injection (LM+). Each dot represents a single cell in the imaging field. Data are from 1 mouse representative of 3 separate experiments. C. Mean Speed (µm/min). D. Straightness Ratio (maximum displacement/track length). E. Displacement Rate (Disp. Rate, µm/min). F. Number of neutrophils per field before (pre LM) and at 15 minutes after *Lm* injection (15 min). Data represents the mean from 3 mice (n = 3). Error bars represent SEM.

To test if these slow moving *Lm* were within neutrophils, we repeated the above experiments with LysM-EGFP knock-in mice [49]. In uninfected mice, neutrophils and monocytes express high and intermediate levels of EGFP, respectively (Figure S2 in Text S1). In the subcapsular RP of uninfected spleens, EGFP^high^ neutrophils were motile in patrolling fashion (Figure 2A–B, Video S2). Upon injection of 10^7^ *Lm*, there was no acute change in crawling speed (15 minutes) or at 2 hours post injection (Figure 2C, Video S2). At 2 hours, neutrophils were significantly more confined as indicated by a lower straightness ratio and displacement rate (p<0.0001, Figure 2D–E). A very small fraction of neutrophils (for example 4 out of over 200 in the imaging field) took up *Lm* (Figure 2, LM+, Video S2). The *Lm* containing neutrophils crawled with an average speed similar to *Lm* negative neutrophils in the same field (Figure 2C). The number of neutrophils in the field increased acutely after *Lm* injection (Figure 2F) and increased in frequency of total splenocytes proportionately to the number of *Lm* injected suggesting recruitment from the bone marrow (Figure S3 in Text S1).

During the course of *Lm* infection the expression level of EGFP in LysM-EGFP mice shifts such that neutrophils and monocytes express similar levels (Figure S2 in Text S1). Thus, we are not able to distinguish neutrophils and inflammatory monocytes in infected mice and will refer to all EGFP positive cells as myelomonocytes (MMCs).

### DC and MMC dynamics at the site of foci of *Lm* growth

**Figure 3:**
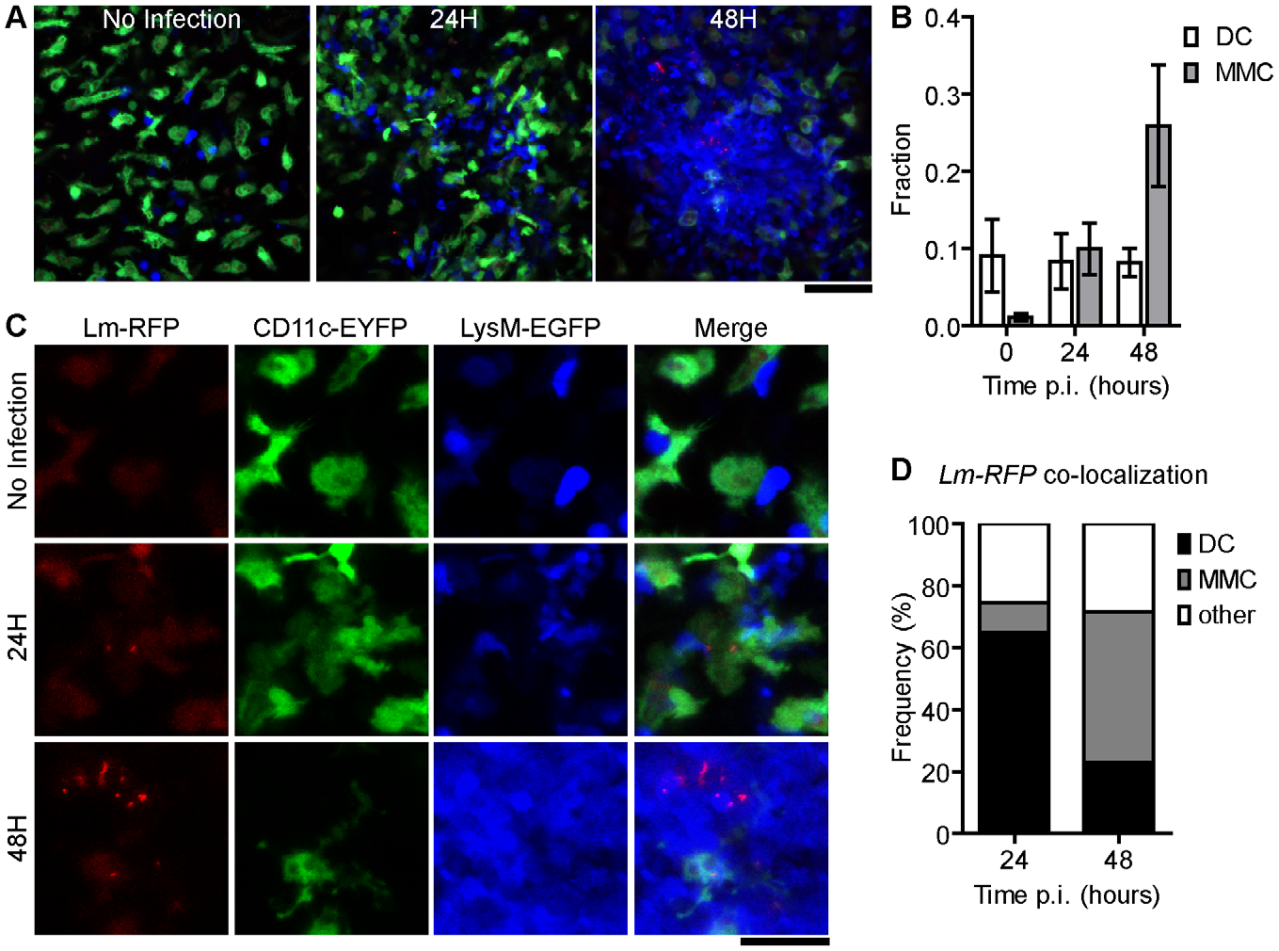
Foci of *Lm* initiate at DC and spread to aggregated MMC. A. Snapshots of intravital images from CD11c-EYFP and LysM-EGFP double transgenic mice at the indicated times p.i. with 2.5×10^4^ *Lm-RFP*. A. Merged images from *Lm-RFP* (red), CD11c-EYFP (green) and LysM-EGFP (blue). Scale bar = 50 µm. B. Fraction of MMC or DC per field (number of positive pixels/total pixels) at the indicated time p.i. C. 2× higher magnification of snapshots in A with single color panels. Scale bar = 25 µm. D. Frequency of co-localized fluorescence of *Lm-RFP* and the indicated cell type. Data is from 3 mice (n = 3). Error bars represent SEM.

For long-term visualization of bacteria, we generated *Lm* that express TagRFP from the *actA* promoter (*Lm*-*RFP*), which is only expressed upon entry into the cytosol (Materials and Methods). To visualize DC and MMC dynamics at the site of *Lm* growth (*Lm* foci), we crossed CD11c-EYFP to LysM-EGFP transgenic mice. Prior to *Lm* injection LysM-EGFP^+^ cells crawled through the RP and displayed transient interactions with scDC (Video S3). Upon *Lm-RFP* infection, MMC accumulated at sites of *Lm-RFP^+^* foci and accumulation increased from 24 to 48 hours (Figure 3A–B). Interestingly, *Lm-RFP* were detected primarily in DC at 24 hours but spread to neighboring cells, including MMC, at 48 hours p.i. (Figure 3C–D).

Although we did not directly observe cell to cell spread, *Lm-RFP* were detectable at the tips of EYFP^+^ DC extensions or “listeriopods”, a mechanism by which *Lm* spread to neighboring cells [50] (Figure S4 in Text S1, Video S4). We also noted fine EYFP^+^ tubules between well-separated scDC (Figure S4 in Text S1, Video S4) that may represent the *in vivo* counterparts of membrane nanotubes [51]. Infected scDC (and non-DC) contained multiple *Lm* (Figure S4 in Text S1, Video S4). Most scDC in the field were enlarged and contained large vacuoles consistent with activation. Several scDC were clustered together with trapped or internalized MMCs (Figure S4 in Text S1).

### MMC aggregation at *Lm* foci is mediated by directed motility

**Figure 4:**
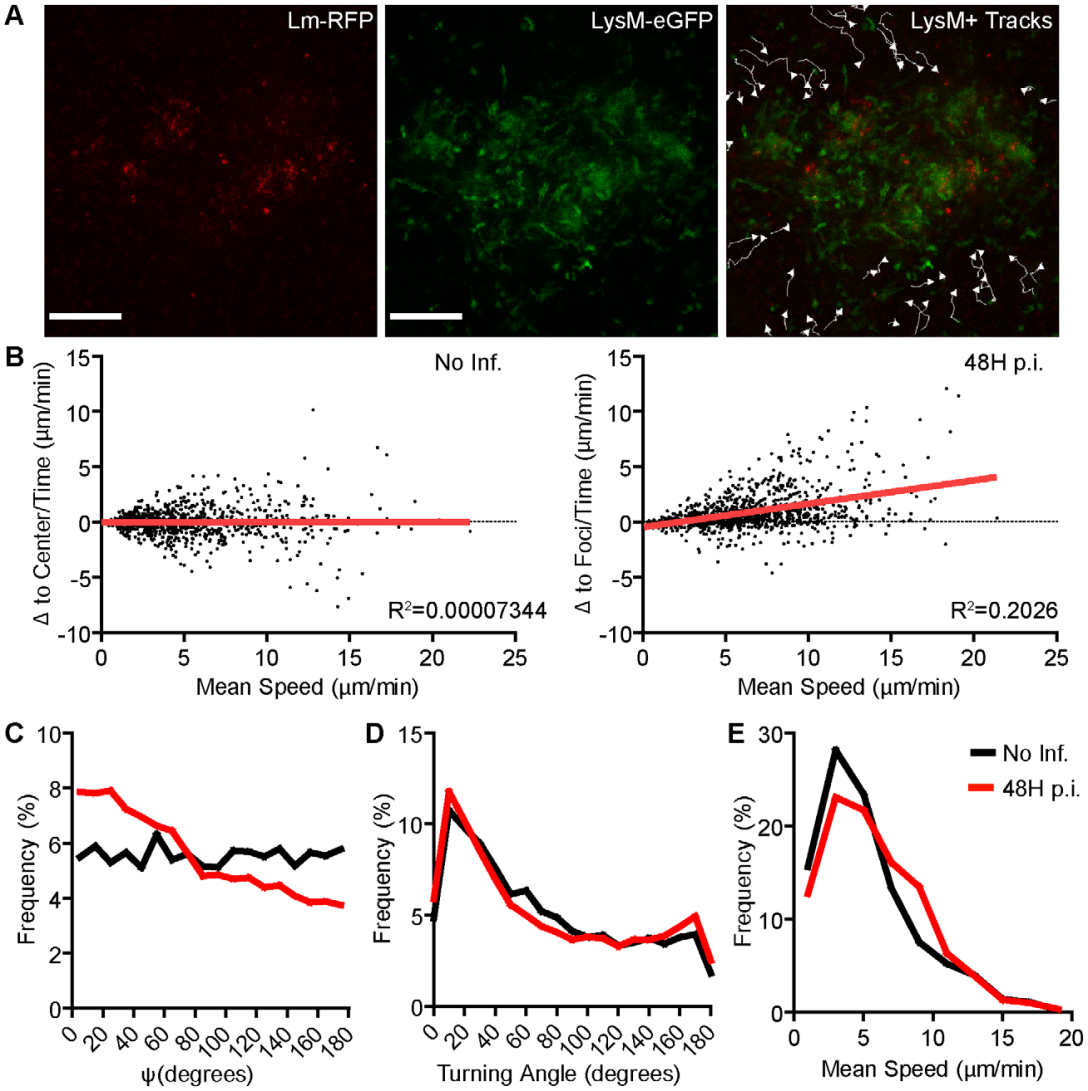
MMC aggregation at *Lm* foci is mediated by directed motility. LysM-EGFP cells were tracked moving from the periphery of *Lm* foci at 48 hours p.i. A. Images from intravital time-series with tracks of LysM-EGFP^+^ cell movement. Scale bar = 100 µm. B. Movement relative to the center of the imaging field (Δ to center/time, µm/min, No Inf.) or to the center of *Lm* foci (Δ to foci/time, µm/min, 48H p.i.) is plotted relative to the mean speed (µm/min) for each cell. Linear regression (red line) is shown. The slope is significantly non-zero (p<0.0001) at 48 hours p.i. C. Frequency distribution of Psi angles (Ψ, degrees). Psi is the angle between a cell trajectory and the foci center (or image center in uninfected mice). At 48 hours p.i., Psi angles are significantly lower (p<0.0001). D. Frequency (%) of Turning Angles (degrees) of LysM-EGFP^+^ cells. The difference is not significant (p = 0.88). E. Frequency (%) of the mean speed per cell. The difference is significant (p<0.0001). Data is pooled from 3 mice per group. No infection, n = 863; 48 hours p.i., n = 1217.

LysM-EGFP^+^ MMCs that accumulate on *Lm-RFP* foci were CD11b^+^ and Gr-1^+^ (Figure S5 in Text S1). To observe the mechanism by which MMC accumulate at the site of infection, intravital microscopy was initiated in LysM-EGFP mice infected with *Lm-RFP* 48 hours prior. MMCs crawled from the periphery (up to 300 µm from the foci center) toward established foci by directed migration, as illustrated by tracks of moving cells (Figure 4A, Video S5). The biased trajectories of MMCs are shown by plotting the change in distance to the foci center against the mean speed for each cell (Figure 4B). Angles (Ψ) between MMC trajectories and the foci center [52] were more frequently less than 90° indicating directed migration towards the focus while no bias in migration within the RP is observed in the absence of infection (p<0.0001, Figure 4C). Overall turning angles were not different compared to cells crawling in uninfected mice however cells moved slightly faster (Figure 4D–E). The signal to join an established focus seemed dominant over signals from some infected cells (Video S5). MMCs within the foci were quite dynamic, but confined within the region of the foci (Video S5).

### MMC protect the host from bacterial spread and tissue damage in the spleen

**Figure 5:**
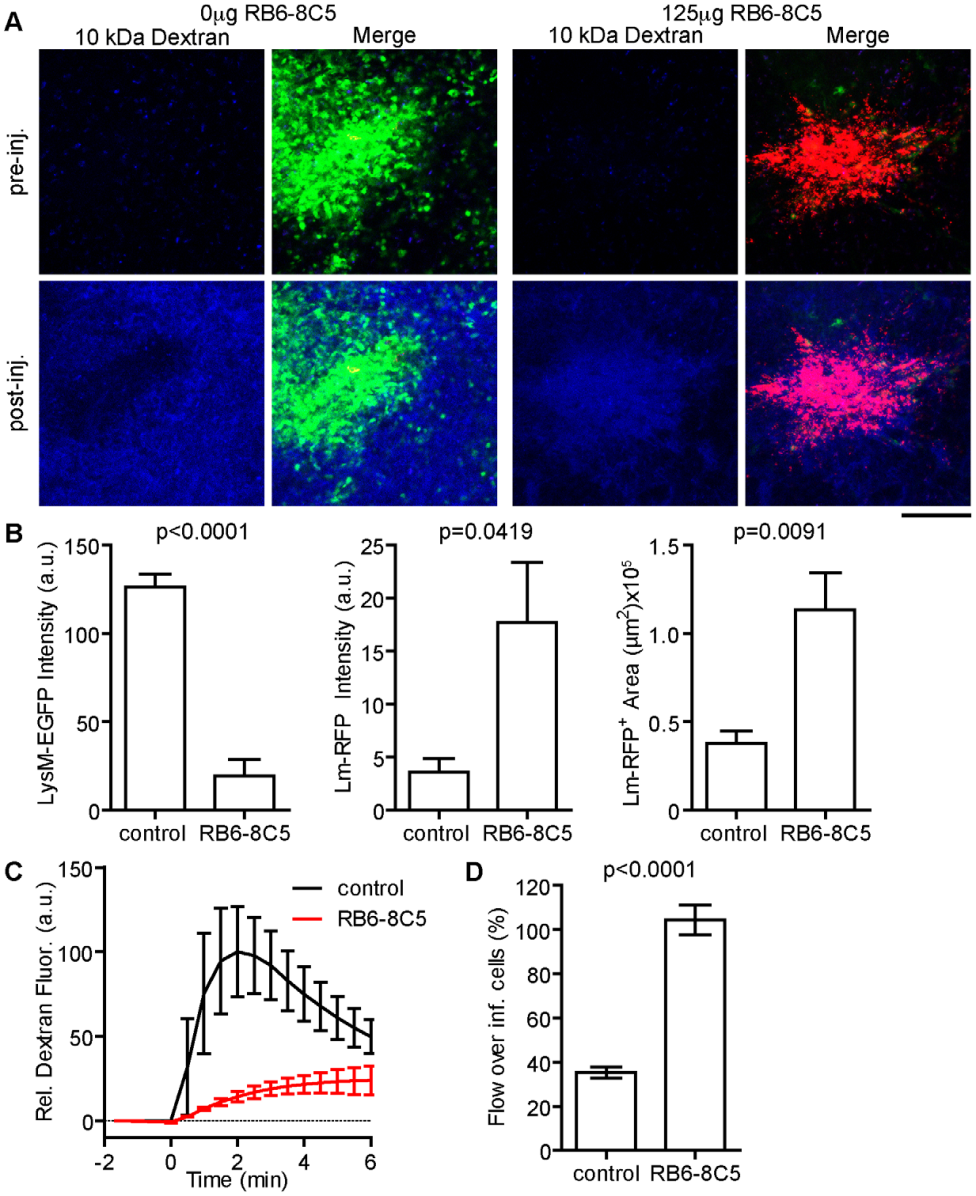
Neutrophils restrict local *Lm* growth and blood flow over infected cells. LysM-EGFP mice were treated or not with 125 µg RB6-8C5 prior to infection with *Lm-RFP*. Their spleens were imaged 48 hours later. Flow rate around the site of infection was determined by injection of Alexa-647 labeled 10 kDa dextran injected i.v. during intravital acquisition. A. Intravital snapshots for each condition before and after dextran injection showing LysM-EGFP^+^ cells (green), *Lm-RFP* (red) and labeled dextran (blue). Scale bar = 100 µm. B. Mean Fluorescence intensity of the indicated signal at the site of *Lm-RFP* foci. C. Average blood flow around *Lm-RFP* foci in control (black) and RB6-8C5 treated (red) mice over time (minutes). D. Blood flow at the site of infection expressed as a percent (%) of that in the peripheral field of view. Data is from 3–5 mice per group.

The *Lm* foci observed here are in the blood filled space of the RP. To test if MMC accumulation affects blood flow around infected cells, we selectively depleted Gr-1^hi^ neutrophils alone or neutrophils and inflammatory monocytes with 125 or 250 µg of anti-Gr-1 antibody (RB6-8C5), respectively (Figure 5A–B and Figure S6 in Text S1). Mice were infected with *Lm-RFP* and intravital imaging was performed 48 hours later. In the absence of neutrophils (125 µg RB6-8C5) *Lm-RFP* growth increased, but they remained in foci (Figure 5A–B). Injection of fluorescent dextran transiently highlights blood flow seconds after i.v. injection. The rate of blood flow in the field of view around *Lm* ^+^ foci was greatly reduced in neutrophil depleted mice compared to untreated (Figure 5C, Video S6). In untreated mice blood flow at the foci is reduced to 40% of that outside the *Lm* foci (Figure 5D). Strikingly, in neutrophil depleted mice, blood flow was specifically increased in these foci relative to the surrounding RP (Figure 5C–D). Thus, MMC, particularly neutrophils, are essential for restriction of blood flow in *Lm* foci and maintenance of surrounding tissue health.

### scDC are not required for restriction of bacterial growth

The observation that *Lm-RFP* are detected in CD11c-EYFP DC in the first 24 hours p.i. is consistent with previous reports that CD11c^+^ cells are required to establish infection and were the only cells containing viable *Lm* in the spleen at this time point [7]. DC were also shown to secrete inflammatory cytokines thought to mediate recruitment and activation of innate effectors [8]. To test the requirement for scDC in maintaining established *Lm* foci, we depleted DC at 48 hours p.i. by treating CD11c-DtR transgenic mice with DT [14]. This treatment induces efficient deletion of CD11c^+^ cells (Figure S7 in Text S1) and has also been shown to deplete MZ macrophage populations [53]. DC depletion resulted no significant change in the number of viable *Lm* recovered from the spleen 24 hours later (72 hours p.i.), although we note a modest reduction (Figure S7 in Text S1). The slight decrease in *Lm* burden after DC depletion suggests that, DC continue to serve as a protective niche for bacterial growth at 48–72 hours p.i, consistent with a role in establishing infection. However at these later time points, *Lm* may have spread to neighboring cells of various cell types masking the effect of DC depletion. The lack of increase in bacterial burden demonstrates that DC do not directly contribute innate protection in the 48–72 hours p.i. Although not addressed here, it is important to note that DCs that migrate to the white pulp are required for priming adaptive immunity necessary for later clearing the infection [54].

### *Lm* specific CD8^+^ T cells have reduced motility within *Lm* foci

**Figure 6:**
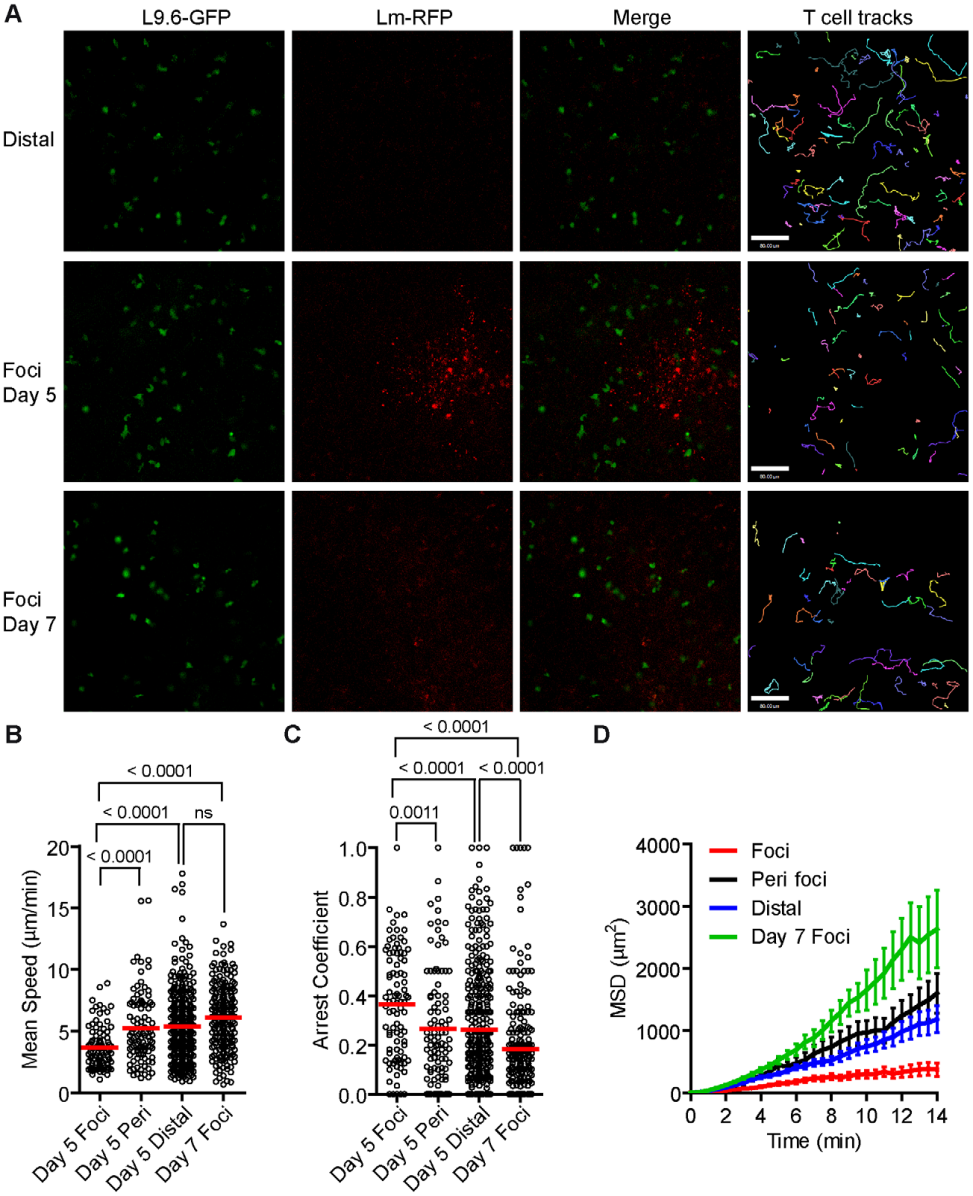
*Lm* antigen specific CD8^+^ T cells have reduced motility within *Lm* foci. One day prior to *Lm-RFP* infection (2.5×10^4^), 0.5 million L9.6-EGFP CD8^+^ T cells were transferred to CB6/F1 recipients. Spleens of infected mice were imaged by intravital microscopy 5 and 7 days p.i. A. Snapshots from intravital time-lapse images including tracks of L9.6-EGFP CD8^+^ T cell movement. Scale bar = 80 µm. B. Mean Speeds (µm/min) of L9.6-EGFP CD8^+^ T cell inside *Lm-RFP* foci (Foci), at the periphery of the foci (Peri foci) and at least 200 µm away from visible foci (Distal). C. Arrest Coefficient (fraction of time a cell crawls <2 µm/min). D. Mean Squared Displacement (MSD µm^2^) over Time (minutes). Error bars represent the SEM. Data is pooled from 3–4 mice per group (Foci, n = 92; Peri foci, n = 99; Distal, n = 336, Foci day 7, n = 205).

On days 3–5 p.i. *Lm-RFP* numbers in the spleen were constant and chronic infection would develop in the absence of an adaptive T cell response [Figure S8 in Text S1, 55]. To track CD8^+^ T effectors at *Lm* foci by intravital microscopy, even after multiple cell divisions, we bred mice with one transgenic allele expressing the L9.6 T cell receptor (TCR) α and β chain and one transgenic allele with EGFP expression driven by the ubiquitin promoter (L9.6-EGFP). L9.6 TCR recognizes the subdominant, but fully protective, p60_217–225_ peptide presented in a stable complex with H-2K^d^ [56]. Naïve CD8^+^ T cells were isolated from L9.6-EGFP mice and transferred to CB6/F1 recipients one day prior to infection with *Lm-RFP*. Excess antigen specific precursors leads to altered CD8^+^ T cell responses [57], and thus we titrated down the number of L9.6-EGFP T cells required to detect T cells at *Lm* foci on day 5 p.i. (Figure S9 in Text S1). At 0.5 million L9.6-EGFP T cells transferred, we detected over 10 T cells at *Lm* foci. At 10 and 100 fold lower transfer numbers, T cells behaved in a similar manner but were too rare for satisfactory statistical analysis (Figure S9 in Text S1). *Lm-RFP* were detected in discrete foci on day 5 p.i., but RFP signal was largely degraded on day 7 consistent with live *Lm* cultured from the spleen at those times (Figure 6A and S8 in Text S1). On day 5 p.i., L9.6-EGFP T cells were detected in close proximity to *Lm-RFP^+^* infected cells (Figure 6A). Inside the foci, T cells were moving slowly (mean speed = 3.64 µm/min) and made extensive contacts with *Lm-RFP^+^* cells (Figure 6B, Video S7). Many L9.6-EGFP cells engaged in contact with the same infected cell throughout the imaging period of up to 30 minutes. Outside the foci, L9.6-EGFP cells proximal (within the field of view of approximately 200 µm from the foci center) and distal (over 200 µm) to the foci moved at a similar speed of 5.23 and 5.37 µm/min, respectively, which was significantly faster compared to cells inside foci (Figure 6B). L9.6-EGFP cells inside the foci displayed significantly higher arrest coefficients (Figure 6C) and were more confined compared to cells outside the foci (Figure 6D). On day 7 p.i., few RFP^+^ bacteria were detected at sites of infection (marked by necrotic and auto-fluorescent tissue) and correlated with fewer live bacteria cultured from the spleen (Figure S8 in Text S1). Coincident with the decrease in live bacteria, L9.6-EGFP CD8^+^ T cell motility was equivalent of those crawling outside the site of infection (Figure 6B–D). Thus, L9.6-EGFP CD8^+^ T cell crawling was restricted when proximal to *Lm-RFP^+^* infected cells but not distal to infected cells and not after live bacteria are killed.

### Specific peptide induces acute arrest in crawling T cells outside *Lm* foci

**Figure 7:**
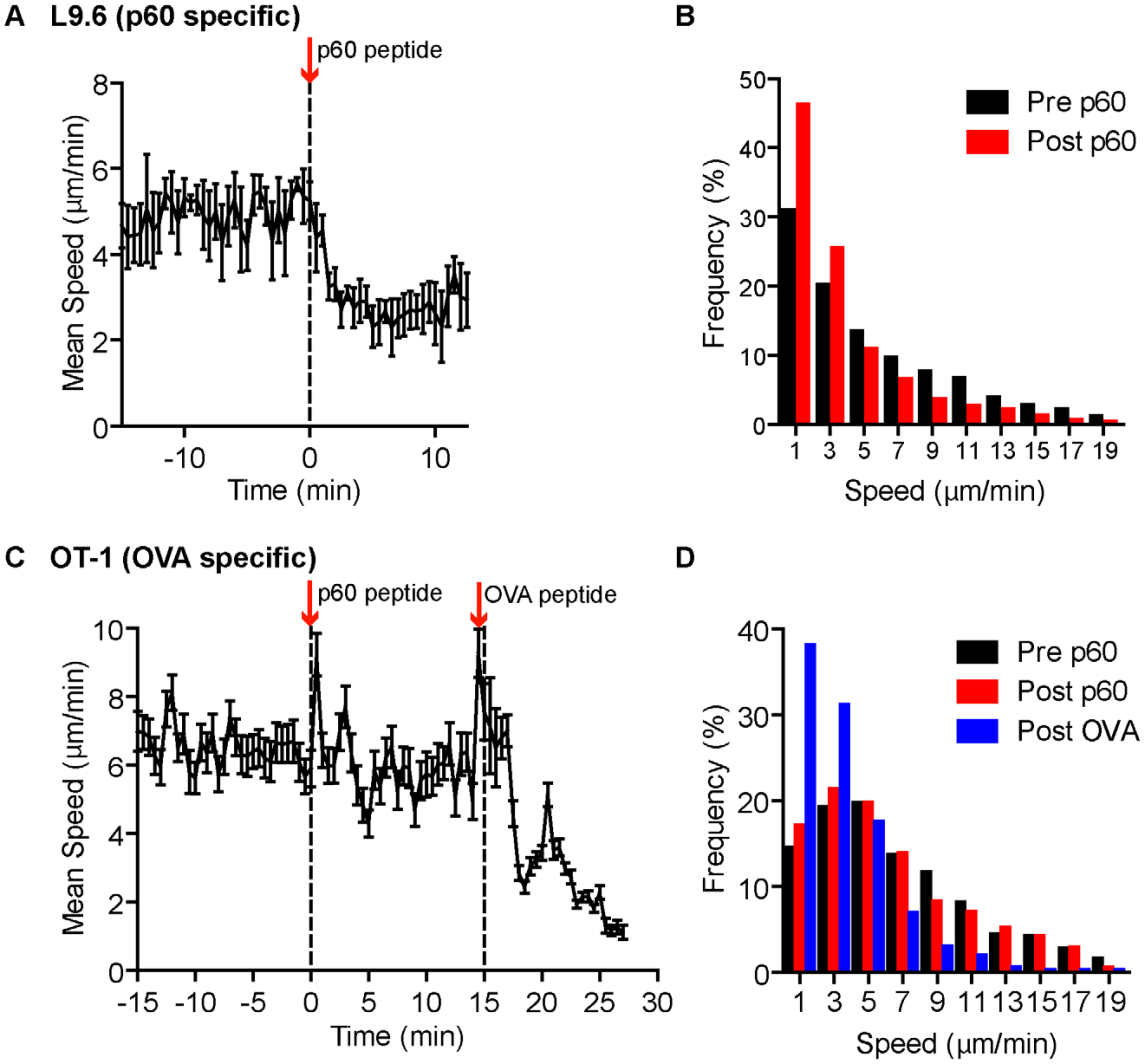
Systemic cognate peptide induces acute arrest of crawling CD8^+^ T cells outside *Lm* foci. One day prior to *Lm-RFP* or *Lm-OVA* infection (2.5×10^4^), 0.5 million L9.6-EGFP or 500 OT-1-dsRed CD8^+^ T cells, respectively, were transferred to recipient mice. Spleens of infected mice were imaged by intravital microscopy 5 days p.i. T cell speeds were monitored outside *Lm* foci before and after acute injection of either p60_217–225_ or OVA_257–264_ peptide. A–B. L9.6-EGFP. C–D. OT-1-dsRed. A, C. Mean speed (µm/min) over Time (minutes) relative to p60_217–225_ peptide injection. Arrows and dotted lines indicate time of injection. Error bars represent the SEM. B, D. Frequency distribution of instantaneous speeds 15 minutes before (Pre) and at least 12 minutes after (Post) peptide. Data is pooled from 3 and 2 mice, n = 119 and 80 for L9.6-EGFP and OT-1-dsRed, respectively.

To test if MHC class I antigen recognition pathway was capable of arresting L9.6-EGFP cell migration outside foci, we injected the specific p60_217–225_ peptide i.v., while monitoring crawling behavior of T cells in extra-foci RP of infected mice. As a control, OT-1-dsRed CD8^+^ T cells were monitored in separate mice. Fluorescent dextran was included with peptide injections to mark the time of arrival in the RP, which was ∼15 seconds after injection. The p60 peptide reduced the average speed of L9.6-EGFP cells from 5.6 to 3.5 µm/min in 1.5 minutes from arrival (Figure 7A,B, Video S8) and had no effect on OT-1 T cell speed (Figure 7C,D, Video S8). OT-1 T cells decelerated in response to injection of OVA_257–264_ (Figure 7C,D, Video S8). These results indicate that antigen-presenting cells in the proximal and distal tissue around the foci can induce antigen specific T cell deceleration if sufficient antigen is available.

### Antigen specific interactions induce confined movement and arrest within *Lm* foci

**Figure 8:**
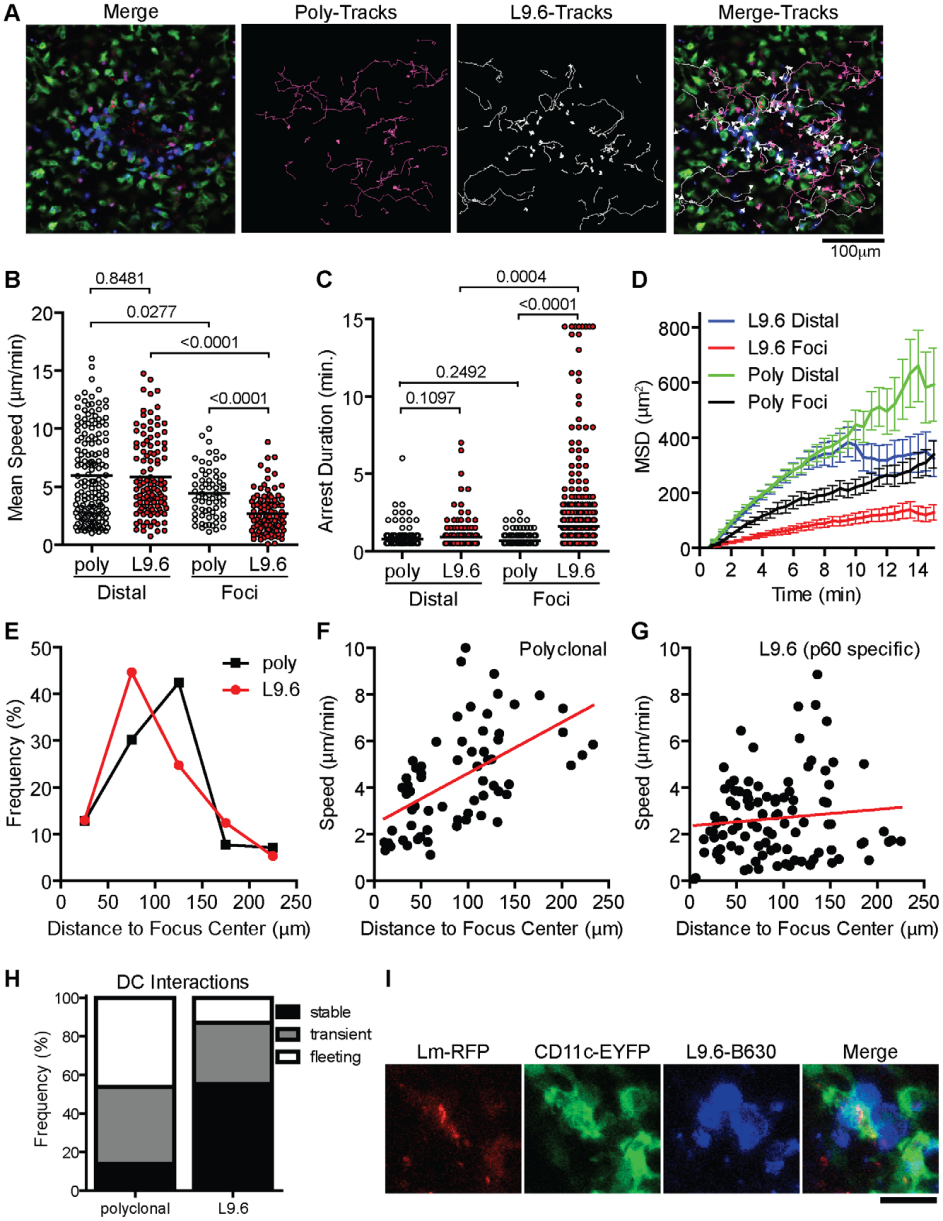
CD8^**+**^ T cell motility inside *Lm* foci is controlled by antigen dependent and independent mechanisms. In vitro activated L9.6-EGFP and polyclonal CD8^+^ T cells were labeled and transferred to CB6/F1 CD11c-EYFP mice at 48 hours post *Lm-RFP* infection (2.5×10^4^) and imaged 24 hours later (72 hours p.i.). A. Intravital snapshot of an *Lm-RFP* focus showing L9.6-EGFP (blue), Polyclonal (magenta), DC (green), *Lm-RFP* (red) and tracks of T cell movement. Scale bar = 100 µm. B–D. Motility parameters of T cells at *Lm-RFP* foci (Foci) or at least 200 µm away from foci center (Distal). B. Mean Speed (µm/min). C. Arrest Duration (consecutive time a cell crawls <2 µm/min). D. Mean Squared Displacement (MSD µm^2^) over Time (minutes). Error bars represent the SEM. E. Frequency (%) of L9.6 (red circles) or polyclonal (black squares) T cells at the indicated distance from the center of *Lm* foci. F–G. Mean Speed (µm/min) relative to distance (µm) from the center of *Lm* foci. H. T cell interactions with DC at *Lm-RFP* foci were characterized as stable (speeds<5 µm/min and within 5 µm of DC), transient (speeds 5–10 µm/min and come within 5 µm of DC) or fleeting (speeds>10 µm/min and come within 5 µm of DC). I. Intravital snapshot of L9.6 T cell and DC interaction. Scale bar is 20 µm. Data is pooled from 2 mice (distal poly, n = 162; distal L9.6, n = 112; foci poly, n = 65; foci L9.6, n = 101).

In order to test if increased arrest in the foci is antigen dependent we activated polyclonal and L9.6 CD8^+^ T cells in vitro, differentially labeled them with Bodipy-630 or Snarf-1 and transferred them to CD11c-EYFP CB6/F1 mice infected with *Lm-RFP* 48 hours prior. This allowed us to simultaneously image antigen specific and polyclonal T cells together with DC and *Lm-RFP*. Including polyclonal cells acts as an internal control for antigen specific affects on motility. Both polyclonal and antigen specific T cells were detected at the site of infection (Figure 8A, Video S9). Crawling speeds of both polyclonal and antigen specific cells were reduced compared to cells distal to the site of infection, however antigen specific cell speeds were further reduced compared to polyclonal cells (Figure 8B). L9.6 T cells displayed increased arrest duration (consecutive time crawling speed is <2 µm/min) and moved in a confined space as shown by the mean squared displacement (µm^2^) over time (Figure 8C–D). Antigen specific L9.6 T cells were also retained closer to the site of infection (Figure 8E). The non-antigen specific effects at the site of infection were reduced as cells were located further away from the foci (Figure 8F) however the effects of antigen seemed to persist further out as antigen specific L9.6 T cells were arrested even 200 µm away from the foci center (Figure 8G). Antigen specific arrest away from *Lm-RFP* ^+^ infected cells may be due to infection of cells below the level of RFP fluorescence detection or acquisition of *Lm* antigens by phagocytic cells for cross-presentation. Next, T-DC interactions were characterized as stable (speeds<5 µm/min and within 5 µm of DC), transient (speeds 5–10 µm/min and come within 5 µm of DC) or fleeting (speeds>10 µm/min and come within 5 µm of DC), polyclonal T cell interactions were mostly fleeting, whereas L9.6 interactions were mostly stable (Figure 8H). At *Lm-RFP* foci we observed multiple L9.6-EGFP cells clustered around individual DC present at the site of infection (Figure 8I). Some DC were infected with multiple *Lm-RFP* while in others no *Lm-RFP* could be detected (Video S10). Interactions between L9.6-EGFP cells and scDC were extensive as DC wrapped their dendrites around T cells and the T cells frequently remained attached to one area on the DC for the entire imaging period (up to 1 hour).

As an additional control for antigen specific effects at *Lm* foci, we used a mutant strain *Lm-218S*, in which a mutation in the p60 amino acid sequence prevents loading of the p60_217–225_ peptide onto class I, but spares p60 function [58]. Because the response to p60_217–225_ is sub-dominant, it can be eliminated without altering the kinetics of bacterial clearance [58]. Because *Lm-218S* is not fluorescent, we identified infectious foci with propidium iodide (PI), which stains nucleic acids accessible in dead cells and neutrophil nucleic acid nets [59], [60]. In vitro-activated L9.6-EGFP cells were labeled with Bodipy-630 and transferred to CD11c-EYFP CB6/F1 mice 48 hours p.i. (Figure S10A in Text S1). L9.6 CD8^+^ T cells were detected at a higher frequency close to *Lm-RFP* foci compared to *Lm-218S* foci (Figure S10B in Text S1), suggesting antigen specific T cells were retained at foci. The average L9.6-EGFP cell speed and displacement rate was decreased significantly in both *Lm-RFP* and *Lm-218S* foci as compared to outside the foci (Figure S10C–D in Text S1). However, T cell motility was further reduced in *Lm-RFP* foci relative to *Lm-218S* foci (3.2 vs. 5.5 µm/min, p<0.0001), demonstrating antigen dependent differences in the speed and confinement of crawling cells at the foci. This is further supported by decreased straightness index and higher arrest coefficients in *Lm-RFP* foci relative to *Lm-218S* foci (Figure S10E–F in Text S1).

## Discussion

We anticipated that our intravital analysis would require confirming some results that are known from earlier studies, but also allow us to break new ground in understanding the dynamics of immune responses in the spleen RP. Our results confirm earlier studies regarding the role of CD11c^+^ cells in initial capture of *Lm* [7] and the importance of MMC in the control of *Lm* growth [9], [10], [11]. We break new ground in demonstrating the speed of *Lm* capture, the dynamic process of directed migration of MMC to the focus, the role of MMC in reducing blood flow in foci, the relatively minor role of CD11c^+^ cells in ongoing innate control of *Lm* in the foci, and the demonstration of antigen independent and dependent components that affect CD8^+^ T cell movement in foci. Some would define DC based on their migration to T cell zones where they present antigen to naïve T cells, while others would include cells that reside in peripheral tissues and promote pathogen clearance by stimulating local cytokine secretion and T cell proliferation [25], [26], [27], [28]. Our results suggest that scDC are bona fide DC by the later inclusion criteria and are phenotypically similar to cells recently described to be essential for activating cytokine production by effector cells in the dermis [25]. Similar DC networks exist in the intestines, kidney and brain where these cells may largely act *in situ*, rather than migrating to secondary lymphoid tissues [31], [61], [62].

DC are responsible for initiating both innate and adaptive immune responses to *Lm* in the spleen [8], [14], but the function of the scDC networks was previously unknown. Our result that *Lm* associate with scDC immediately after injection is consistent with DC depletion studies that show CD11c^+^ cells are required to capture *Lm* in the spleen [7]. Extension and retraction of DC dendrites was observed before and after interaction with *Lm*. The continued environmental probing even after associating with *Lm* may enhance interactions with immune cells such as neutrophils, inflammatory monocytes, natural killer (NK) cells, memory T cells or CTLs that can contribute to innate or adaptive recognition of infection. One day after infection, scDC contained live, intracellular bacteria (as detected by *actA* driven RFP expression) and *Lm* displayed mechanisms for cell-to-cell spread, such as listeriopods. Depletion of CD11c^+^ cells at 48–72 hours p.i. eliminated the *Lm* ^+^ DC and moderately decreased the number of viable *Lm* in the spleen. This suggests that scDC may serve as a reservoir for bacteria and that *Lm* may exploit the scDC niche to maintain the infectious foci. Consistent with this, it was previously shown that elevated numbers of DC in the spleen resulted in increased bacterial load [63]. Infection of DC may be beneficial to the host as these cells orchestrate both innate and adaptive immune responses [8], [14]. Although DC are required to prime adaptive cells required for clearing *Lm*, our data also suggest that once this role is fulfilled the persistently infected scDC in the red pulp are not necessary for innate control of infection. However, the scDC may have already fulfilled their roles at the time of deletion by early recruitment of MMC, which can then take over bacteriacidal and antigen presenting functions [64].

MMC were required for control of *Lm* as previously described [9], [10], but are not required to initiate or maintain foci through the first 48 hours of infection. This contrasts with *Leishmania major* infection in the skin, in which neutrophils are the reservoir required to establish infection [34]. These results also contrast with the role of macrophages in granulomas of zebrafish [37] in which motile macrophages promote spread of infection. The chemotactic attraction of MMC to *Lm* foci may serve to reduce egress of infected cells and dissemination to other organs. NK cell and inflammatory monocyte recruitment to *Lm* infected cells in the spleen is dependent on signals from chemokines [8]. However, recently it was shown that recruitment of monocytes to *Lm* foci in the liver was more dependent on adhesion molecules and not chemokines [12]. Thus, MMC recruitment mechanisms are likely to depend on the cell types infected and the specific structure of the tissue microenvironment. At *Lm* foci, swarming of MMCs around foci is reminiscent of responses to *Toxoplasma gondii* infection in the LN [52]. However, MMC swarming and mingling with infected scDC did not result in acute disruption of infected cells as was observed as for CD169^+^ macrophages in the LN. In contrast, DC internalized MMC at the site of infection. MMC at the site of infection may be a source of antigens important for priming adaptive responses [18]. Swarming and aggregation of MMCs at *Lm* foci was protective and maintained integrity of surrounding tissue. The MMC dependent restriction of blood flow in the foci may serve to wall off potentially harmful inflammation as well as spread of bacteria. Densely packed phagocytic cells surrounding the infected cells may act as a secondary containment to prevent bacterial escape from foci.

Antigen specific effector CD8^+^ T cells infiltrated *Lm* foci and made prolonged interactions with infected cells. The speeds of L9.6-EGFP cells in the spleen RP were similar to effector T cells in the lymph nodes and peripheral sites such as skin [40], [65], [66], [67], [68]. The average speed of T cells within *Lm-RFP* ^+^ foci (day 5 p.i.) was significantly reduced, but cells were quite dynamic with some forming asymmetric mobile junctions or “kinapses” and others forming more stable non-motile symmetric contacts that may represent immunological synapses [69]. Indeed, L9.6 T cells engaged infected cells with prolonged arrest durations over 10 minutes which is sufficient time to perform CTL activity [23]. However, we did not observe any obvious cytolysis of infected cells, which may indicate other mechanisms of clearance such as anti-microbial activity stimulated by IFNγ [21], [22]. Interestingly, antigen specific cells resumed migration coincident with clearance of live bacteria. This suggests that antigen presentation does not persist at the site of infection in the absence of live bacteria. The introduction of systemic cognate antigen acutely arrested crawling T cells suggesting TCR-pMHC interactions do induce a robust “stop” signal to CD8^+^ T cells. Previous reports show that CD4^+^ T cells moved more rapidly (∼6 µm/min) in liver granulomas of BCG infected mice with few T cells arrested [38]. *Mycobacterium tuberculosis* (TB) organisms grow slowly and induce delayed CD4^+^ T cell responses [70]. The relatively faster movement to CD4^+^ T cells in liver granulomas compared to CD8^+^ T cells in *Lm* foci in the spleen may reflect the relatively poor direct presentation of TB or BCG antigens. The comparison of polyclonal and antigen specific T cells, as well as use of “antigen null” *Lm* allowed us to finely dissect out antigen dependent and independent affects on motility at the site of infection. Antigen independent components that reduce T cell movement in *Lm* foci of the spleen may include reduced oxygenation/nutrient supply due to poor blood flow, increased cellular congestion, changes in extracellular matrix and increased expression of adhesion molecules like ICAM-1 on infected cells [12], [71]. Recruitment of non-specific T cells may induce local crowding and prevent productive interactions between specific T cells and DC [72]. In a recent study, recall responses to *Lm* resulted in clusters of antigen specific and non-specific memory CD8^+^ T cells around infected cells in the RP within 6 hours and both specific and non-specific cells generated IFNγ, whereas only antigen specific memory cells made CCL3 [73]. It will be interesting to determine if non-specific effector cells are also stimulated to make IFNγ in *Lm* foci in the RP in primary responses. However, our data show that recruitment of non-specific T cells is transient as only antigen specific T cells were retained at foci in the effector phase of the primary response. Hierarchic antigen specific and antigen independent affects on motility are also observed in CD8^+^ and CD4^+^ T cells recruited to sites of *Leishmania donovani* infection in the liver and *Leishmania major* infection in the ear dermis, respectively [39], [42].

In a recent study, ex vivo spleen fragments were imaged to study T cell and DC interactions during priming in *Lm* infection in the WP [16]. Imaging spleen sections allows access to the WP. In contrast, we imaged the intact spleen where blood flow was maintained [45], but limited our observations to the RP and the effector arm of the immune response. Preservation of blood flow was integral to several findings in this study: a) ability to visualize the fate of *Lm* immediately after injection; b) observing the recruitment of MMC from the blood, which occurred continuously throughout the infection; c) and that MMC accumulation in foci excludes blood flow potentially ‘walling-off’ the infection from the rest of the host. Our observations suggest a complex relationship between *Lm* and spleen DC where DC may provide a niche for pathogen growth but at the same time mediate protection by recruitment of innate effectors and presenting antigens to effector T cells.

## Materials and Methods

### Ethics statement

This study was carried out in strict accordance with the recommendations in the Guide for the Care and Use of Laboratory Animals of the Public Health Service (National Institutes of Health). The protocol was approved by the Institutional Animal Care and Use Committee of the New York University School of Medicine (Assurance of Compliance Number: A3435-01). All surgery was performed under Ketamine, Xyalazine and Acepromazine anesthesia, and all efforts were made to minimize suffering.

### Mice

LysM-EGFP, a gift of Dr. T. Graf, CD11c-EYFP mice, a gift of Dr. M. Nussenzweig, and CD11c-DtR, a gift of Dr. D. Littman, on the C57/B6 background were maintained in a colony at the barrier facility of the Skirball Institute of Biomolecular Medicine at New York University (New York, NY). CB6/F1 recipients were purchased from Jackson Labs (Bar Harbor, ME). L9.6 on a Balb/c background and Ub-GFP mice on a C57/B6 background were crossed to generate L9.6-EGFP CB6/F1 donors. CD11c-EYFP homozygous mice on the C57Bl6 background were bred to Balb/c mice to generate CD11c-EYP CB6/F1 recipients. OT-1 and Actin-dsRed mice were purchased from Jackson and crossed to generate OT-1-dsRED. *Lm* infected mice were housed under animal BSL2 conditions in a special room of the Skirball Institute specific pathogen free facility.

### Fluorescent *Lm-RFP* (DP-L 5538)

*Lm* strains were constructed in the DP-L4056 strain background [74]. TagRFP from *Entacmaea quadricolor* [75] was codon optimized for expression in gram positive bacteria with Gene Designer software [76] and the cDNA was synthesized de novo (DNA2.0, Menlo Park, CA). The synthetic gene was cloned downstream of the *actA* promoter in the vector pPL2 and stably integrated at the *tRNA* ^Arg^ locus of the bacterial chromosome in the as described previously [74]. All molecular constructs were confirmed by DNA sequencing.

### Bacteria culture and inoculation of mice

Virulent *Lm* strain aliquots were kept at −80°C and grown in Brain Heart Infused media (BHI, Fisher) for 3–4 hours until ∼0.1 optical density (OD) at 600 nm. *Lm* were diluted to the appropriate concentration in 200 µl PBS for inoculation into mice. For acute imaging experiments 1×10^7^ Bodipy-630 labeled bacteria were injected into the retro-orbital plexus during image acquisition. For all other time points (24 hours and later) 2.5×10^4^ bacteria were injected. To facilitate location of infectious foci, in some experiments mice were infected with a 10-fold higher infectious dose (2.5×10^5^ *Lm-RFP*).

### MMC and DC depletion in mice

For MMC depletion, LysM-EGFP mice were treated with 125 or 250 µg RB6-8C5 antibody by i.p. injection 5 hours prior to infection with *Lm-RFP*. Assays were performed 48 hours p.i. For DC depletion, C57/B6 and CD11c-DtR transgenic mice were infected with *Lm-RFP* and 48 hours later treated with 1 µg DT by i.p. injection. Assays were performed 72 hours p.i. (24 hours after DT treatment). Images of the spleens were taken by intravital micrcoscopy and then splenocytes were collected for FACS or lysed with 0.05% Triton-X 100 and bacteria were plated in serial dilutions on Brain heart infused (BHI) agarose plates to obtain colony counts.

### Cells

Naïve CD8^+^ T cells were isolated from spleen by CD8^+^ T cell negative selection kit (Miltenyi Biotec, Auburn, CA). 0.5×10^6^ L9.6-EGFP or 500 OT-1-dsRed naïve cells were adoptively transferred to CB6/F1 and CD11c-EYFP CB6/F1 or C57/B6 recipients by i.v. retro-orbital injection in 100–200 µl PBS, one day before infection.

### In vitro T cell activation

1×10^6^ negatively selected naïve CD8^+^ T cells were incubated with 2.5×10^7^ APC (ACK treated and irradiated spleen cell suspension) in 20 ml OK-DMEM, 10% fetal bovine serum with 10 nM p60_217–225_ and supplemented with 25 U/ml recombinant IL-2 in T25 flask (Corning 430372 or BD 353081). On day 4 post-activation, medium was replaced with fresh media plus 25 U/ml recombinant IL-2 and expanded up to 50 ml in T75 flask (BD 353135). T cells were used on day 6 post activation.

### Bacteria and cell labeling

In vitro activated T cells were labeled with 1 µM Bodipy 630/650 methyl bromide (B22802, Invitrogen, Carlsbad, CA) or 1 µM Snarf-1 (S22801, Invitrogen, Carlsbad, CA) by incubation at a concentration of 10–20×10^6^ cells per ml in PBS at 37°C for 15 minutes. *Lm* were labeled at a concentration of 0.5–2×10^8^ CFU per ml of 5 µM Bodipy 630/650 methyl bromide in BHI at 37°C for 15 minutes. After labeling, cells or bacteria were washed 2–3 times with PBS.

### Immunostaining

Cell preparations for FACS were prepared by mashing the spleen through 40 µm filters in FACS buffer. Red blood cells were lysed by ACK. Frozen sections were prepared by fixing tissue fragments with 4% PFA PBS for 1 hour on ice and perfused with 30% sucrose PBS at least until tissue sank to the bottom of solution. Cells and tissue were stained with the following antibodies from eBioscience (San Diego, CA): CD11c (N418)-APC, CD11b (M1/70)-APC, MHC class II (M5/114.15.2)-APC, F4/80 (BM8)-APC, Ly-6C (AL-21) Pe-Cy7, Ly-6G (RB6-8C5) Alexa-700.

### Peptide injections

10 µl of 10 µM p60_217–225_ peptide or 10 µg of OVA_257–264_ peptide plus 4 µg Alexa-647 10 kDa dextran (D-22914, Molecular Probes) were diluted into 100 µl PBS and injected i.v. into the retro-orbital plexus of anesthetized mice during image acquisition. Alexa-647 10 kDa dextran was included to mark the time of injection.

### Surgical preparation for spleen intravital imaging

Mice were anesthetized with an intraperitoneal injection of Ketamine (50 mg/kg), Xyalazine (10 mg/kg) and Acepromazine (1.7 mg/kg) and boosted with a half dose every 30–60 minutes. The spleen was externalized by making a 1 cm incision just below the ribcage. The organ was gently tethered out of the body and a custom made plastic apparatus slid between the spleen and the mouse body. The apparatus was used to keep the organ out of the body and aids in stability. The apparatus does not disrupt the vasculature or connective tissue of the spleen. The mouse was then laid on a stage with the spleen positioned over a cover slip. The stage and mouse were heated to 37°C by flowing heated air over the system. The mouse was covered to prevent drying out the tissue and overheating. Oxygen was delivered to a mask that covers the snout to ensure the animal, and tissue, receive adequate oxygen. To verify that blood circulation through the spleen was not disrupted by the procedure, 4 µg Alexa-647 10 kDa dextran in 100 µl PBS were routinely injected i.v. into the retro-orbital plexus during image acquisition.

### Confocal intravital microscopy

For intravital imaging in spleen, we used a Zeiss LSM 510 or 710 laser scanning confocal microscope (Carl Zeiss, Thornwood, NY) using an inverted Plan-Apochromat 20×/0.75, 25×/0.8, 40×/1.3 Oil DIC, or FLUAR 40×/1.3 Oil objectives. EGFP, RFP and Alexa-647 10 kDa Dextran were imaged using appropriate combinations of 488-nm, 546-nm, and 633-nm laser lines and BP 505–530, BP 560–615, and LP 650 filter sets, respectively. ECFP, EGFP and EYFP were imaged using combinations of 458-nm, 488-nm and 514-nm laser lines and BP 475–525, BP 505–530 and LP 530 filter sets, respectively. Time-lapse images were acquired by scanning 20×460.7×460.7 (20×) or 20×230.3×230.3 (40×) µm at 30 second intervals. In some cases z- stacks of images were taken at 3×10 µm steps or images were tiled during acquisition using a motorized stage. For high-resolution images of scDC and *Lm*, z- stack images were taken at 1 µm steps covering 14 to 20 µm.

### Motility analysis and statistical calculations

Movement of cells in tissue was tracked using Volocity software (Improvision, Waltham, MA). Only cells that remained in the field of view for more than 5 frames (2.5 minutes) out of a total at least 30 frames (15 minutes) were counted as crawling cells. Distribution of cell velocities and motility parameters were non-Gaussian and thus Mann-Whitney rank sum test was used to compare data from each group. Statistical calculations and graphing were done in Prism (GraphPad Software, LaJolla, CA).

### Mouse Genome Database (MGD) accession ID numbers

LysM (Lyz2) MGI:96897, LysM-eGFP (Lyz2^tm1.1Graf^) MGI:2654931, CD11c (Itgax) MGI:96609, CD11c-eYFP (Tg(Itgax-Venus)1Mnz) MGI:3835666, CD11c-DTR (Tg(Itgax-DTR/EGFP)57Lan) MGI:3057163, CD11b (Itgam) MGI:96607, MHC class II (H2-Ab1) MGI:103070, F4/80 (Emr1) MGI:106912, Gr-1 (Ly6g) MGI:109440, Ly-6C (Ly6c1) MGI:96882, CD8 (Cd8a) MGI:88346, TCR α (Tcra) MGI:98553, TCR β (Tcrb) MGI:98578. Mouse Genome Informatics, The Jackson Laboratory, Bar Harbor, Maine. World Wide Web (URL: http://www.informatics.jax.org).

## Supporting Information

Text S1 Supplemental Figures. Supplemental figures cited in the main text.
(9.17 MB DOC)

Video S1 Real time injection of labeled *Lm* during image acquisition in the spleen of CD11c-EYFP mice. 10^7^ Bodipy-630 labeled *Lm* were injected i.v. into the retro-orbital plexus while acquiring intravital images in the spleen of CD11c-EYFP mice. CD11c-EYFP^+^ cells are shown in green, Bodipy-630 labeled *Lm* are shown in red. The time series is shown first as the entire field of a 14 µm z-stack collapsed to show all slices. The following time series is a 3D reconstruction of the same data set zoomed to a *Lm* and DC interaction. Next, 3D reconstructions of z-stack images are shown in rotation for 3 examples of *Lm* and DC pair. Z-stacks were taken at 1 µm steps during intravital imaging. Scale bar lengths are labeled in each series. Video corresponds with data from Figure 1.
(4.14 MB MOV)

Video S2 Real time injection of labeled *Lm* during image acquisition in the spleen of LysM-EGFP mice. Bodipy-630 labeled *Lm* were injected i.v. into the retro-orbital plexus while acquiring images in the spleen of LysM-EGFP mouse. At 2 hours post injection *Lm* (red) were detected within motile LysM-EGFP^+^ cells (green). Video corresponds with data from Figure 2.
(2.76 MB MOV)

Video S3 Development of *Lm-RFP* foci in the spleen of CD11c-EYFP and LysM-EGFP double transgenic mice. Intravital time series were taken to observe *Lm-RFP* (red), CD11c-EYFP^+^ DC (green) and LysM-EGFP^+^ MMC (blue) interactions before, 24 or 48 hours post infection (p.i) with 2.5×10^4^ *Lm-RFP*. At 24 hours p.i., *Lm-RFP* were primarily inside DC (solid circles) or tethered to cells, likely in listeriapods (dotted circle). At 48 hours p.i., MMCs accumulated on infected cells and only a small fraction of *Lm-RFP* were inside DC (arrow). Video corresponds with data from Figure 3.
(4.71 MB MOV)

Video S4 CD11c-EYFP^+^ DC and LysM-EGFP^+^ MMC dynamics at *Lm-RFP* foci. Intravital time series were taken to observe *Lm-RFP* (red), CD11c-EYFP^+^ DC (green) and LysM-EGFP^+^ MMC (blue) interactions at 24 or 48 hours p.i with 2.5×10^5^ *Lm-RFP*. At 24 hours p.i., multiple *Lm-RFP* were inside a single DC (closed arrow) or non-fluorescent cells (open arrow). Activated DC with large vacuoles and internalized myelomonocytes were also present at foci (boxed regions). At 48 hours p.i., *Lm-RFP* were detected at the tips of extended DC dendrites or “listeriopods” (closed arrow) and nanotube connection between adjacent DC were apparent (open arrow). Video corresponds with data from Figure S4 in Text S1.
(3.02 MB MOV)

Video S5 LysM-EGFP^+^ MMC home to *Lm-RFP* foci in a directed fashion. At 48 hours p.i. with 2.5×10^5^ *Lm-RFP*, LysM-EGFP^+^ MMC (green) move towards the focus of *Lm-RFP* (red) from the periphery of the imaging field. Video corresponds with data from Figure 4.
(2.86 MB MOV)

Video S6 LysM-GFP^+^ MMC accumulation serves to reduce blood flow around *Lm-RFP* foci. LysM-EGFP mice were treated with 0 or 125 µg RB6-8C5 by intra-peritoneal (i.p.) injection 5 hours prior to infection with *Lm-RFP*. Image acquisition in the spleen was initiated 48 hours p.i. and Alexa-647 dextran (blue) was injected i.v. in the retro-orbital plexus. LysM-EGFP and *Lm-RFP* are shown in green and red, respectively. Video corresponds with data from Figure 5.
(7.08 MB MOV)

Video S7 In vivo activated L9.6-EGFP CD8^+^ T cells have reduced motility within *Lm-RFP* ^+^ foci. Naive CD8^+^ T cells were isolated from L9.6-EGFP mice and 0.5 million were transferred to CB6/F1 recipients. Mice were infected with 2.5×10^4^ *Lm-RFP* one day later. Spleens of infected mice were imaged by intravital microscopy 5 or 7 days p.i. Intravital time course showing L9.6-EGFP (green) at an *Lm-RFP* focus (red) or at least 200 µm from foci of *Lm-RFP* (Distal to *Lm-RFP*). Video corresponds with data in Figure 6.
(4.91 MB MOV)

Video S8 Antigenic p60_217–225_ peptide induces a reduction in speed of *in vivo* activated L9.6-EGFP CD8^+^ T cells outside of *Lm-RFP* foci. One day prior to *Lm-RFP* or *Lm-OVA* infection, 0.5 million L9.6-EGFP (green) or 500 OT-1-dsRed (red) CD8^+^ T cells, respectively, were transferred to recipient mice. Spleens of infected mice were imaged by intravital microscopy 5 days p.i.. T cell speeds were monitored outside the site of infection before and after acute injection of either p60_217–225_ or OVA_257–264_ peptide. Injection of peptide is marked by co-injection of Alexa-647 10 kDa dextran (blue). Video corresponds with data in Figure 7.
(3.59 MB MOV)

Video S9 Deceleration in CD8^+^ T cell speeds at *Lm-RFP* foci is both antigen dependent and independent. In vitro activated and labeled L9.6-EGFP or polyclonal CD8^+^ T cells were transferred to CB6/F1 CD11c-EYFP mice at 48 hours p.i. with 2.5×10^4^ *Lm-RFP* and imaged 24 hours later (72 hours p.i.). Intravital time course in the spleens of infected mice are shown with L9.6-EGFP (blue) and polyclonal (magenta) T cells, CD11c-EYFP^+^ DC (green) and *Lm-RFP* (red). Video corresponds with data in Figure 8.
(3.03 MB MOV)

Video S10 L9.6-EGFP CD8^+^ T cells make extensive contacts with both *Lm-RFP* infected and non-infected scDC at foci of infection. L9.6-EGFP cells were activated and rested in vitro, labeled with Bodipy-630 and transferred to CB6/F1 CD11c-EYFP mice at 48 hours p,i. with 2.5×10^4^ *Lm-RFP*. Intravital time course in the spleens of infected mice are shown with L9.6-EGFP cells (blue), DC (green) and *Lm-RFP* (red). Multiple L9.6-EGFP CD8^+^ T cells interacting with an infected scDC are indicated (arrow). Video corresponds with data in Figure 8 and S9 in Text S1.
(3.62 MB MOV)
