# Supplementary material for: Dynamic Imaging of the Effector Immune Response to Listeria Infection In Vivo
Source: PLoS Pathog. 2011 Mar 24;7(3):e1001326. doi: 10.1371/journal.ppat.1001326 (PMC3063765; doi:10.1371/journal.ppat.1001326)
Supplement: Text S1 — Supplemental Figures. Supplemental figures cited in the main text. (9.17 MB DOC) [file ppat.1001326.s001.doc]

**Supplemental Data**

**Dynamic Imaging of the Effector Immune Response to *Listeria* Infection *in vivo***

**Janelle C. Waite, Ingrid Leiner, Peter Lauer, Chris S. Rae, Gaetan Barbet, Huan Zheng, Daniel A. Portnoy, Eric G. Pamer, and Michael L. Dustin**

**Supplemental Figures**

**
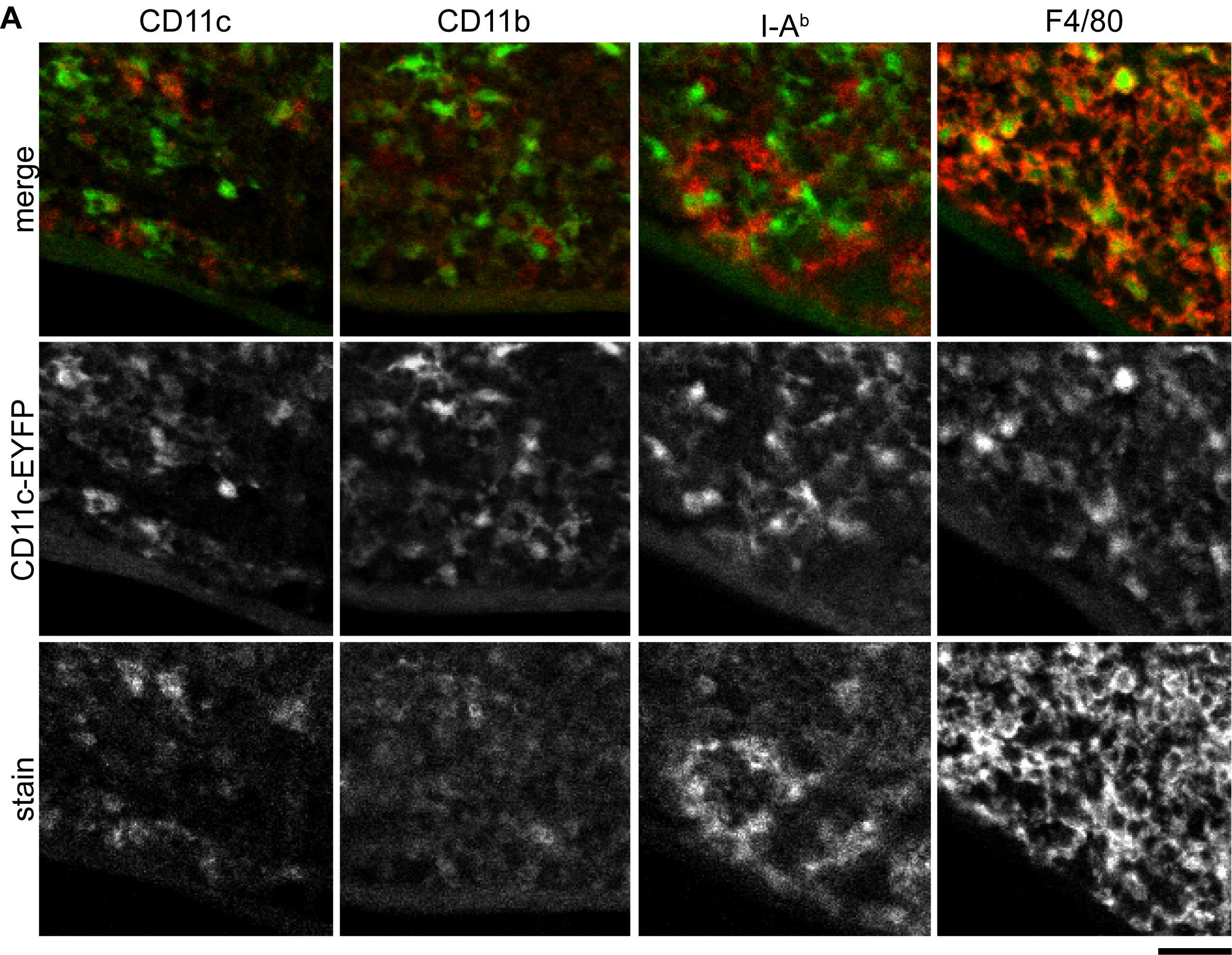
**

**
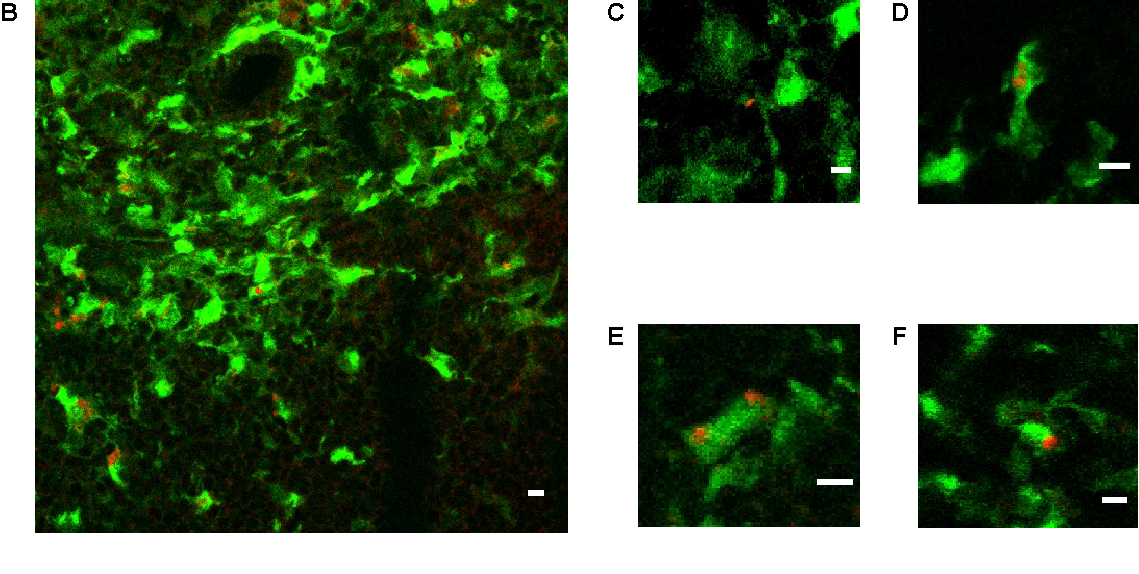
Figure S1. Surface molecule staining of CD11c-EYFP^+^ cells in the scRP and localization of acutely injected *Lm*.** A. Frozen sections of spleen from uninfected CD11c-EYFP mice were stained for the indicated surface molecules. Pseudo-colored merge images are shown with CD11c-EYFP (green) and the indicated stain (red). Pictures were taken near the outer capsule in the same region imaged in intravital microscopy experiments. Scale bar = 25μm. B-F. 10^7^ Bodipy-630 labeled *Lm* (red) were injected in the retro-orbital plexus of CD11c-EYFP (green) mice and spleens were perfusion fixed 5 minutes later. Frozen 10μm sections were cut and mounted onto slides for imaging. B. Whole image showing several *Lm* associated with CD11c-EYFP^+^ cells. C. Cropped image showing single *Lm* associated with a CD11c-EYFP^+^ cell dendrite. D. Cropped image showing a single *Lm* inside a CD11c-EYFP^+^ cell. E. Cropped image showing two *Lm* associated with CD11c-EYFP^+^ cell. F. Cropped image showing a single *Lm* inside a CD11c-EYFP^+^ cell. Scale bar = 5μm. Data is from 2 mice. Data is related to Figure 1.

**
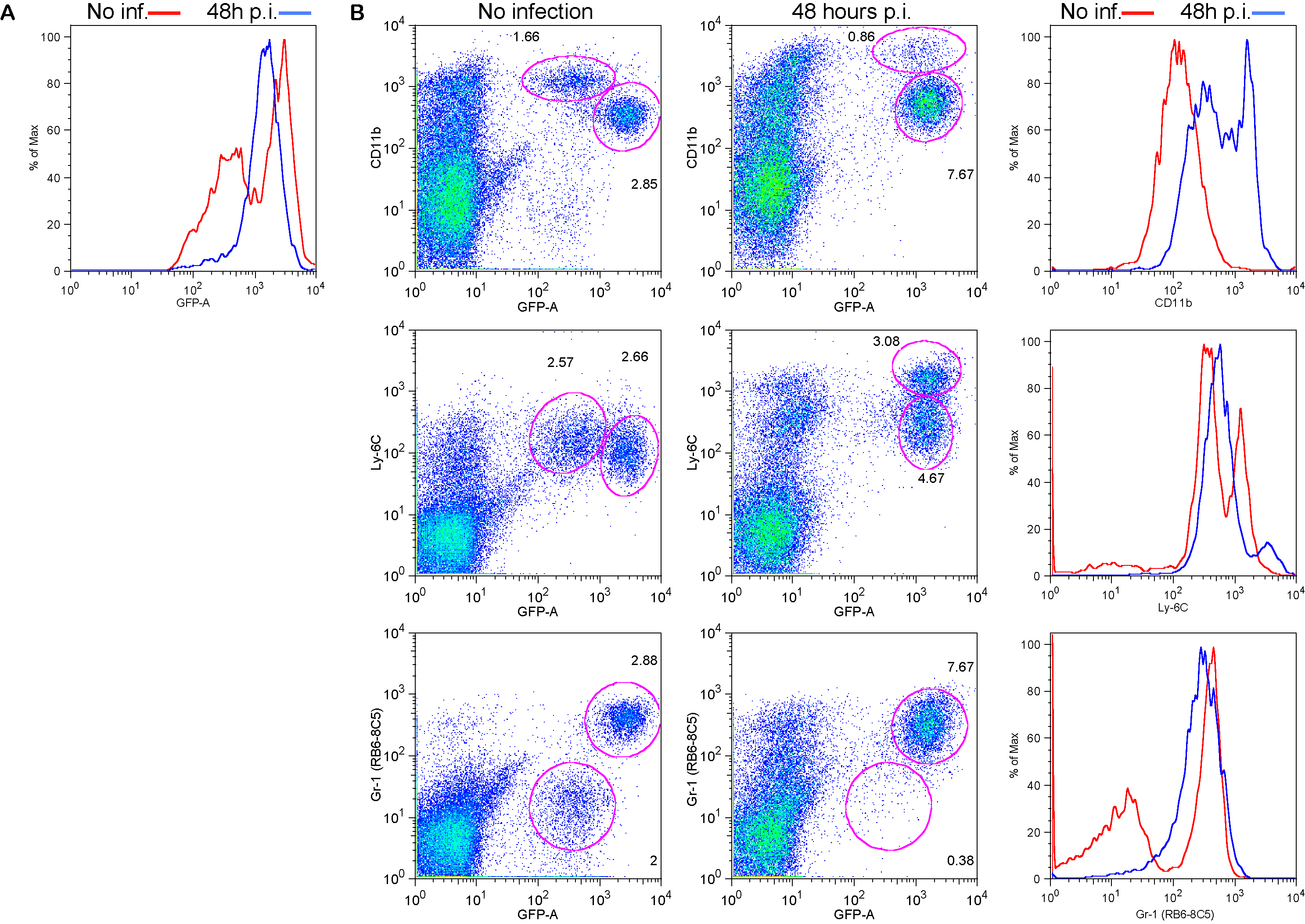
**

**Figure S2. LysM-EGFP expression labels myelomonocytes the spleen.** Splenocytes were isolated from LysM-EGFP mice either uninfected or 48 hours post infection (p.i.). A. GFP^+^ gate showing GFP expression intensity in uninfected (No inf., red) and 48 hours p.i (48h p.i., blue). B. Surface marker staining of the indicated molecules (Y-axis) and GFP expression (X-axis). GFP^+^ cells were gated and histograms of surface marker expression are shown in uninfected (No inf., red) and 48 hours p.i. (48h p.i., blue). Data is related to Figure 2.


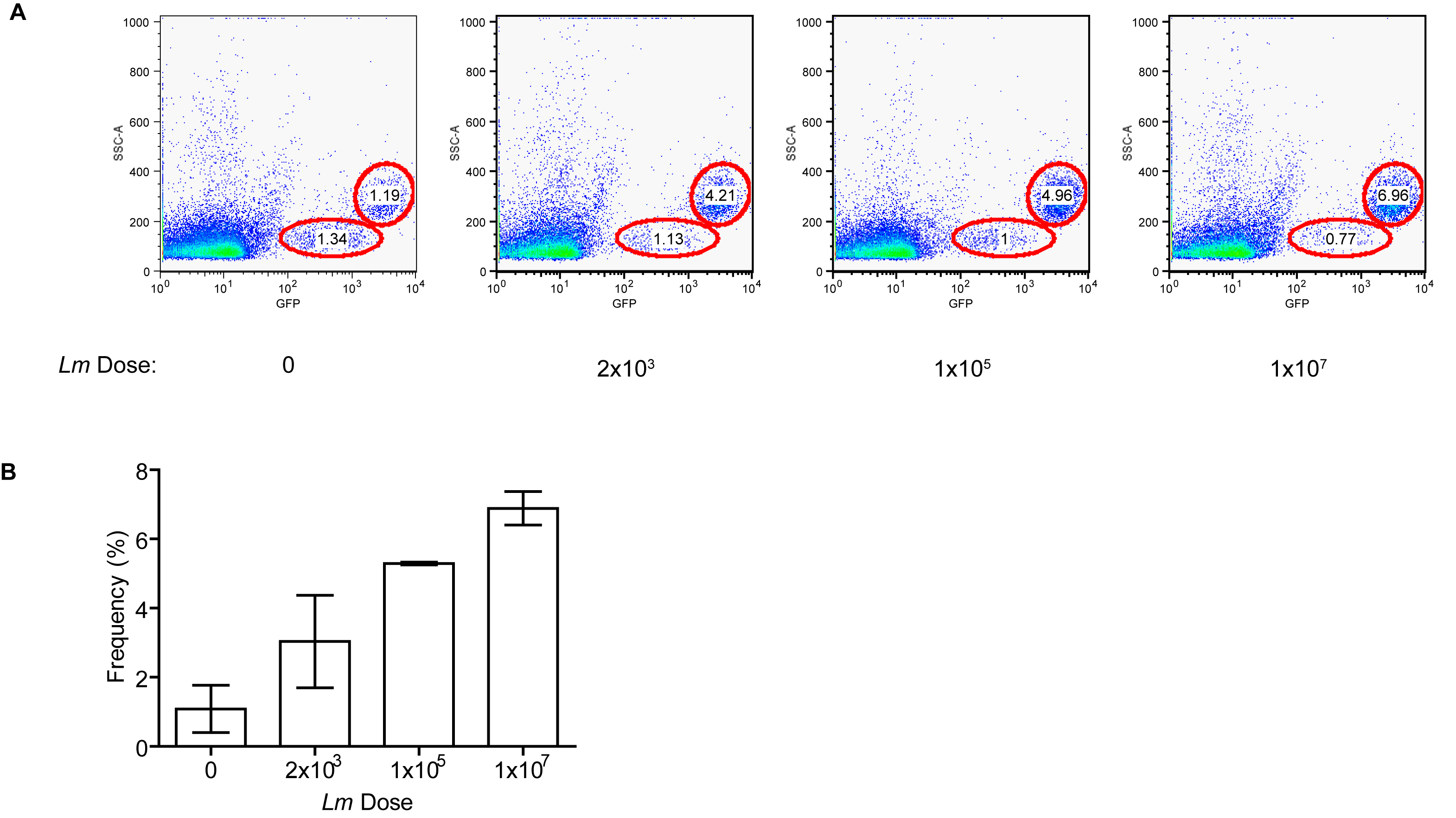


**Figure S3. Neutrophil accumulation in the spleen occurs within 1 hour of *Lm* injection in a dose dependent manner.** LysM-EGFP mice were infected with the indicated number of *Lm* and splenocytes were collected 1 hour later for analysis by FACS. A. GFP intermediate and high populations are gated. B. Quantification of GFP high populations. Data is from 3 mice, Error bars represent SEM. Data is related to Figure 2.

**
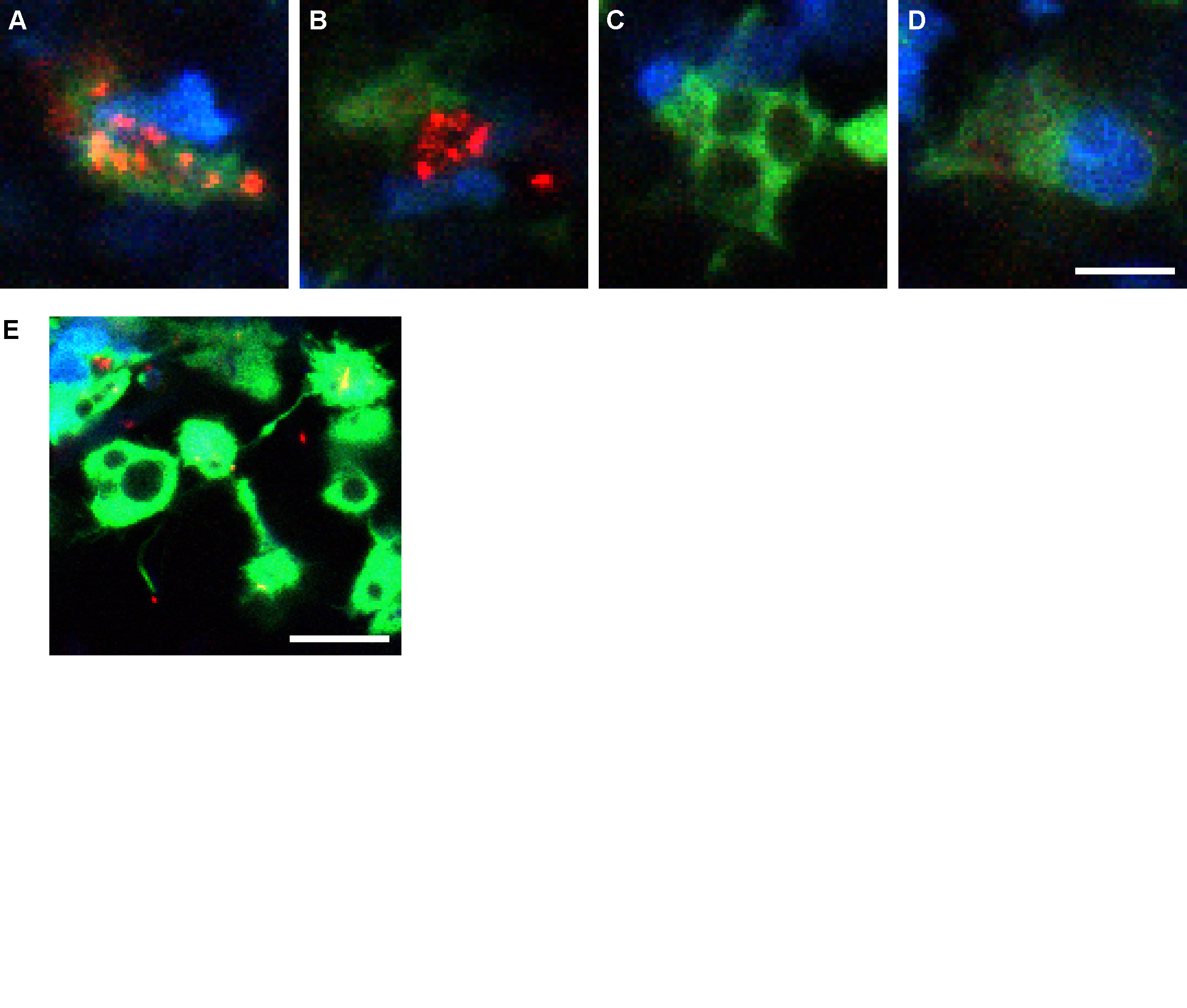
**

**Figure S4. *In vivo* visualization of *Lm-RFP,* MMC and DC at foci of infection.** CD11c-EYFP and LysM-EGFP double transgenic mice were infected with 2.5x10^5^ *Lm-RFP* and their spleens were imaged 24 (A-D) and 48 (E) hours post infection. CD11c-EYFP (green), LysM-EGFP (blue) and *Lm-RFP* (red) signals are shown. A. CD11c-EYFP DC containing multiple *Lm-RFP*. B. Non-fluorescent cell containing multiple *Lm-RFP*. C. CD11c-EYFP cells fused together with large vacuoles. D. LysM-EGFP MMC taken up by CD11c-EFYP DC. E. Listeriopods protrude from infected CD11c-EYFP DC and nanotubes connect adjacent DC. This data is representative of 2-4 fields/mouse from at least 12 mice. Snapshots are from Movie S4. Data is related to Figure 3.

**
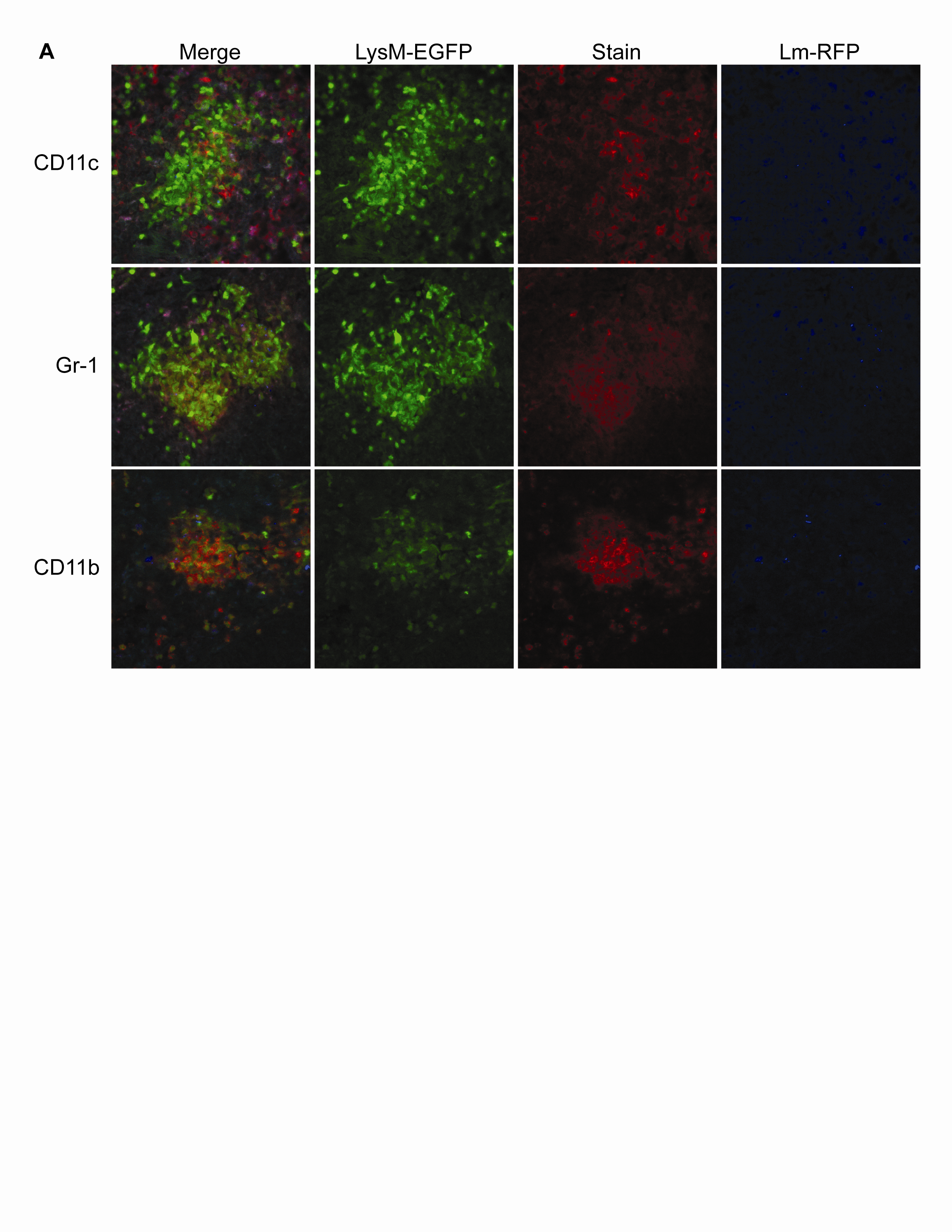
Figure S5. LysM-EGFP^+^ cells at *Lm-RFP* foci are positive for CD11b and Gr-1 but not CD11c.** Frozen sections from spleens of LysM-EGFP mice 48 hours p.i. with *Lm-RFP* stained with indicated fluorescently conjugated antibodies. Panels show merge, LysM-EGFP (green), indicated stain (red) and *Lm-RFP* (blue). Data is related to Figure 4.

RB6-8C5 (μg)

**
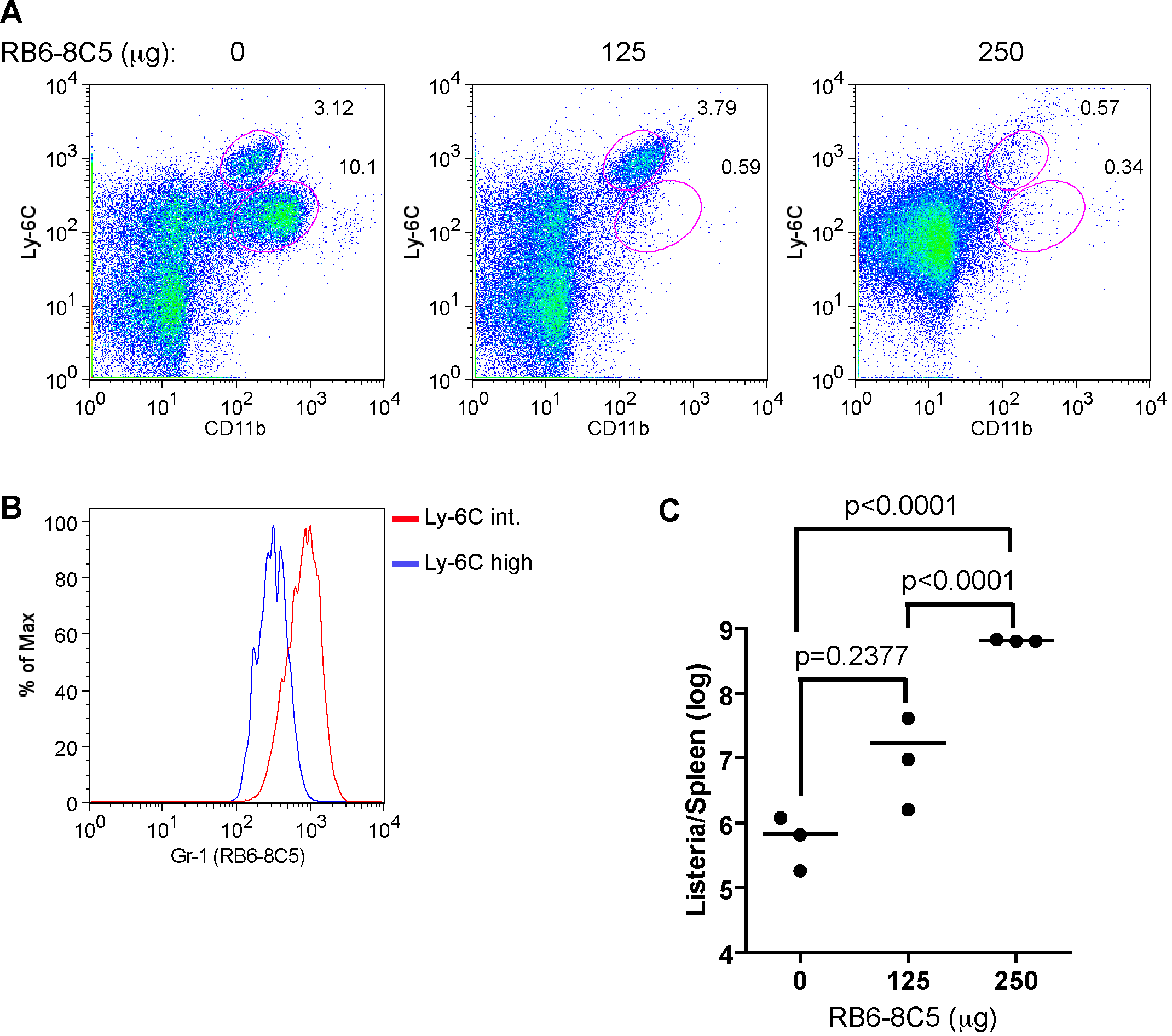
**

**Figure S6. Graded depletion of Gr-1 expressing MMC causes increased *Lm* burden in the spleen 48 hours p.i.** LysM-EGFP mice were treated with RB6-8C5 at the indicated dose by intra-peritoneal (i.p.) injection 5 hours prior to infection with *Lm-RFP*. 48 hours after infection (48h p.i.) splenocytes were isolated for FACS analysis (A-B) or lysed to determine *Lm* burden by colony counts (C). A. FACS analysis of surface marker expression of CD11b (X-axis) and Ly-6C (Y-axis) and gating on Ly-6C high inflammatory monocytes and Ly-6C intermediate neutrophil populations. B. Ly-6C high (blue) and intermediate (int., red) gated populations are plotted for expression of Gr-1 (RB6-8C5). C. Splenocytes were lysed with 0.05% Triton-X 100 and bacteria were plated in serial dilutions on BHI agarose plates to obtain colony counts. from spleens of mice treated with the indicated dose of RB6-8C5. Data is from 3 mice per group. Data is related to Figure 5.


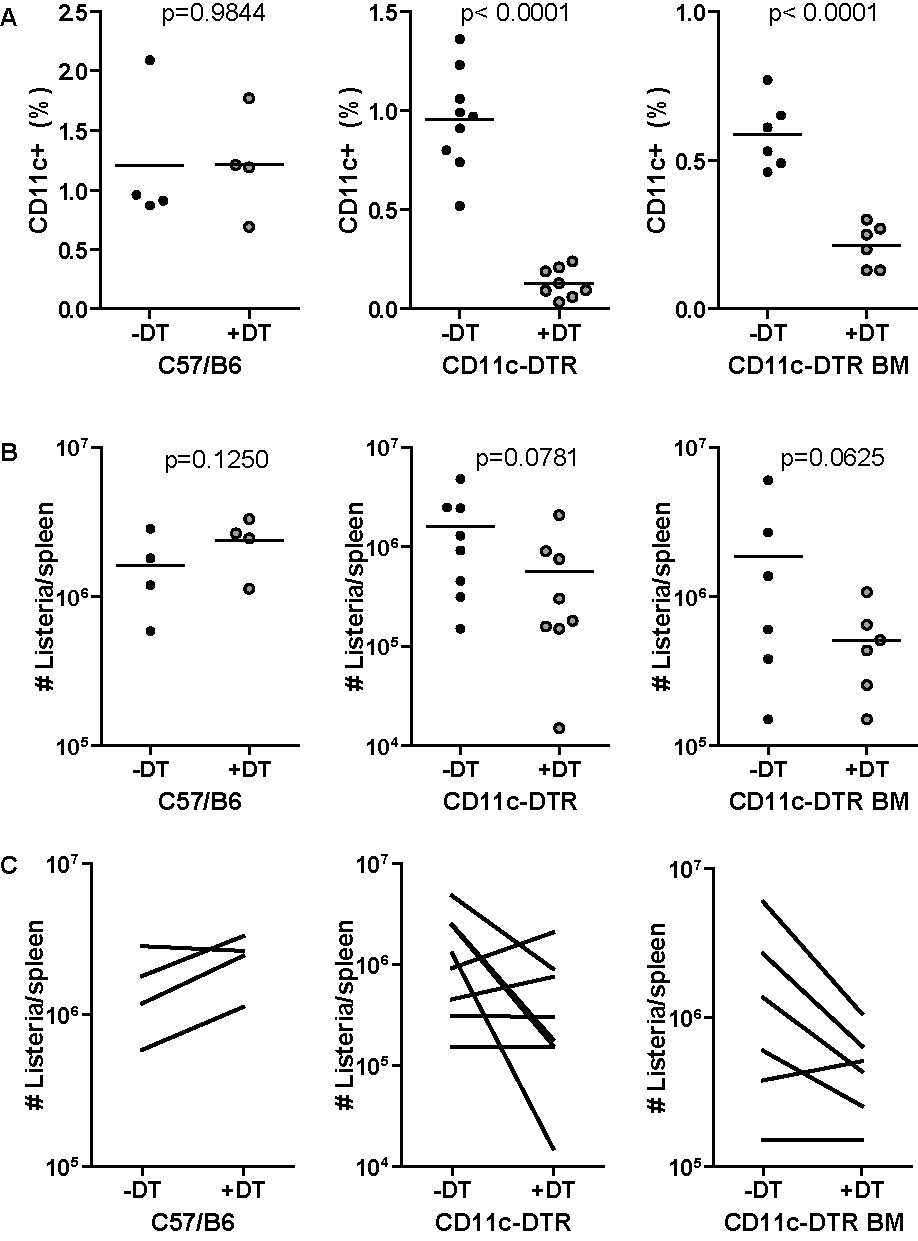


**Figure S7. DC are not required to restrict *Lm* growth in the spleen.** C57/B6, CD11c-DTR or CD11c-DTR bone marrow chimera (CD11c-DTR BM) mice were infected with *Lm-RFP* and 48 hours later treated with 1μg DT by i.p. injection. Analysis was performed 24 hours after DT treatment (72 hours p.i.). A. Splenocytes were isolated for analysis of CD11c expression by FACS. B. Splenocytes were lysed with 0.05% Triton-X 100 and bacteria plated in serial dilutions on BHI agarose plates to obtain colony counts. P values were obtained by Wilcoxon signed rank test. Each dot represents data from one mouse, C57/B6, n=4; CD11c-DTR, n=9; CD-11c-DTR. Data is related to Figure 3-5.


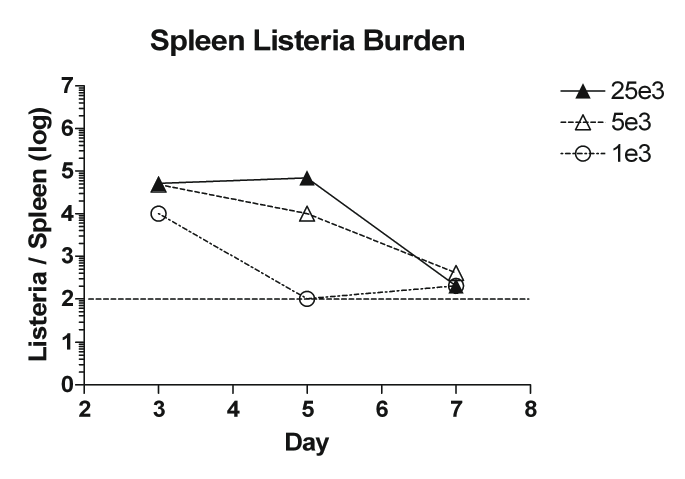


**Figure S8. *Lm-RFP* grow in the spleen up to day 3 and 5 and are cleared on day 7 post infection**. CB6/F1 mice were infected with the indicated dose of *Lm-RFP* and sacrificed at the indicated day post infection. Splenocytes were lysed with 0.05% Triton-X 100 and bacteria were plated in serial dilutions on Brain Heart Infused agarose plates to obtain colony counts. Data is related to Figure 6.


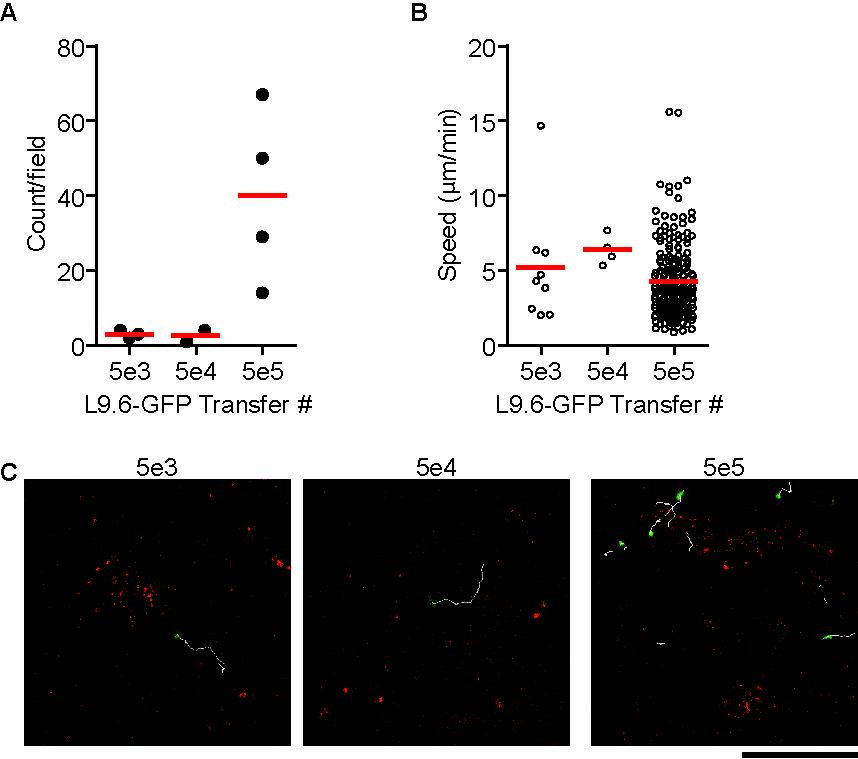


**Figure S9. Titration of the number of L9.6-EGFP T cells transferred in order to detect T cells at *Lm* foci on day 5 p.i.** L9.6-EGFP T cells were transferred at the indicated number to recipient mice one day prior to infection with *Lm-RFP*. A. Count of T cells detected at *Lm* foci on day 5 p.i. Each dot represents the number from individual mice. B. Speeds of T cells at *Lm* foci. C. Snapshots from intravital time-series showing L9.6-EGFP T cells (green), *Lm-RFP* (red) and T cell tracks (white). Scale bar=200μm. Data is related to Figure 6.


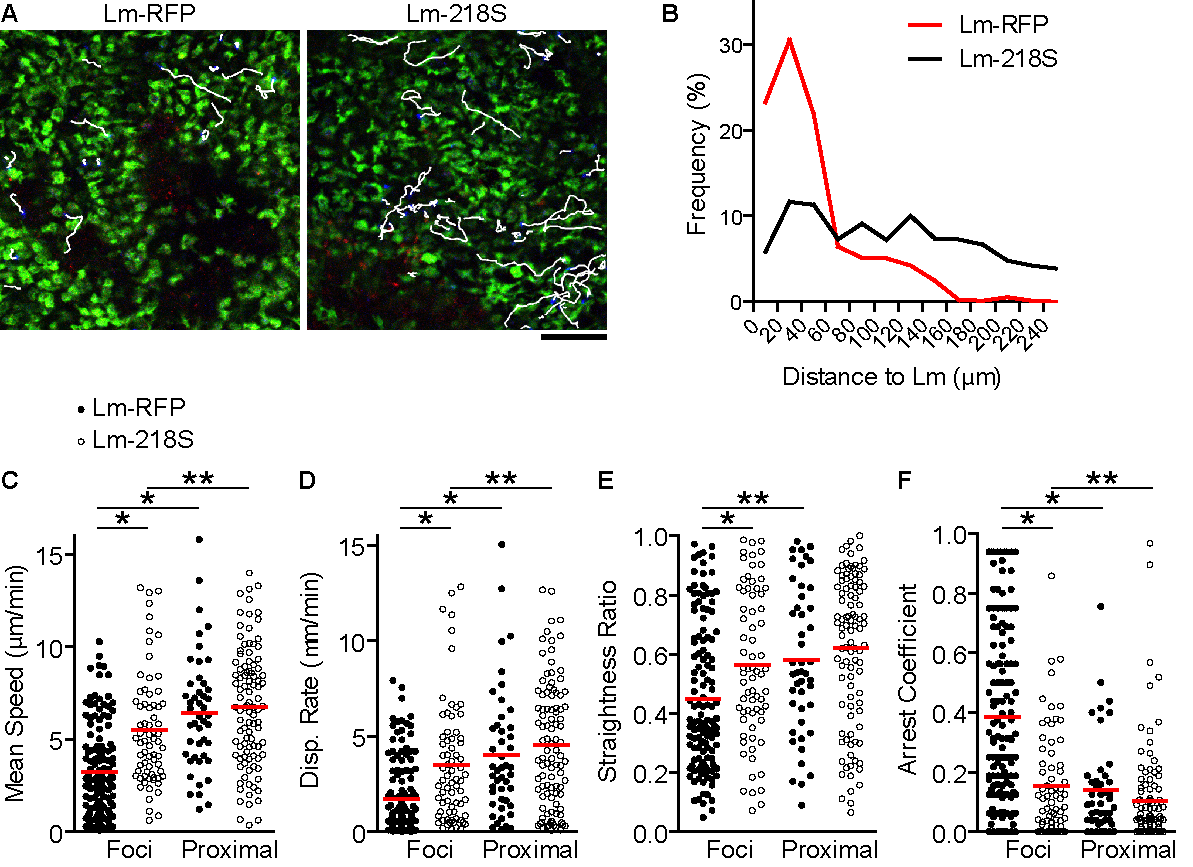


**Figure S10. CD8^+^ T cell motility inside *Lm* foci is controlled by antigen dependent and independent mechanisms.** L9.6-EGFP cells were activated and rested in vitro, labeled with Bodipy-630 and transferred to CB6/F1 CD11c-EYFP mice at 48 hours p.i. with 2.5 x 10^4^ *Lm-RFP* or *Lm-218S*. A. Intravital images in the spleens of infected mice. L9.6-EGFP Tracks (white), L9.6-EGFP cells (blue), DC (green) and *Lm-RFP* (red) or PI staining of *Lm-218S* foci (red) are shown. Scale bar = 90 μm. B. Frequency (%) of L9.6-EGFP cells at the indicated distance from the nearest *Lm* (determined by PI staining in both *Lm-RFP* and *Lm-218S* foci). C-F. L9.6-EGFP motility parameters from mice infected with *Lm-RFP* (filled circles) or *Lm-218S* (open circles). Foci are defined as regions were *Lm-RFP* or PI staining (*Lm-218S*) is detected. L9.6-EGFP cells are characterized as being inside (Foci) or outside the foci but within the viewing field (Proximal). Each circle represents a cell from at least 3 mice per group. C. Mean Speed (μm/min). p<0.0001, *; p=0.0057, **. D. Displacement Rate (μm/min). p<0.0001, *; p=0.0327, **. E. Straightness Ratio (maximum displacement/track length). p=0.0010, *; 0.0024**. F. Arrest Coefficient (fraction of time a cell crawls < 2 μm/min), p<0.0001,*; p=0.0253, **. Data is from 3 mice per group. Data is related to Figure 8.
